# An overview of the past decade of bufalin in the treatment of refractory and drug-resistant cancers: current status, challenges, and future perspectives

**DOI:** 10.3389/fphar.2023.1274336

**Published:** 2023-10-04

**Authors:** Qingmei Ye, Xin Zhou, Han Ren, Fangxuan Han, Rong Lin, Juan Li

**Affiliations:** ^1^ Hainan General Hospital & Hainan Affiliated Hospital of Hainan Medical University, Haikou, Hainan, China; ^2^ Key Laboratory of Tropical Medicinal Resource Chemistry of Ministry of Education, Key Laboratory of Tropical Medicinal Plant Chemistry of Hainan Province, College of Chemistry and Chemical Engineering, Hainan Normal University, Haikou, Hainan, China; ^3^ The Fifth People’s Hospital of Hainan Province & Affiliated Dermatology Hospital of Hainan Medical University, Haikou, Hainan, China; ^4^ Hubei Province Key Laboratory of Traditional Chinese Medicine Resource and Chemistry, Department of Pharmacy, Hubei University of Chinese Medicine, Wuhan, Hubei, China

**Keywords:** refractory cancers, drug resistance, bufalin, overcome, mechanisms

## Abstract

Profound progress has been made in cancer treatment in the past three decades. However, drug resistance remains prevalent and a critical challenge. Drug resistance can be attributed to oncogenes mutations, activated defensive mechanisms, ATP-bind cassette transporters overexpression, cancer stem cells, *etc.* Chinese traditional medicine toad venom has been used for centuries for different diseases, including resistant cancers. Bufalin is one of the bufadienolides in toad venom that has been extensively studied for its potential in refractory and drug-resistant cancer treatments *in vitro* and *in vivo*. In this work, we would like to critically review the progress made in the past decade (2013–2022) of bufalin in overcoming drug resistance in cancers. Generally, bufalin shows high potential in killing certain refractory and resistant cancer cells via multiple mechanisms. More importantly, bufalin can work as a chemo-sensitizer that enhances the sensitivity of certain conventional and targeted therapies at low concentrations. In addition, the development of bufalin derivatives was also briefly summarized and discussed. We also analyzed the obstacles and challenges and provided possible solutions for future perspectives. We hope that the collective information may help evoke more effort for more in-depth studies and evaluation of bufalin in both lab and possible clinical trials.

## Introduction

### Drug resistance remains a significant challenge that undermines effective cancer treatment

**FIGURE 1:**
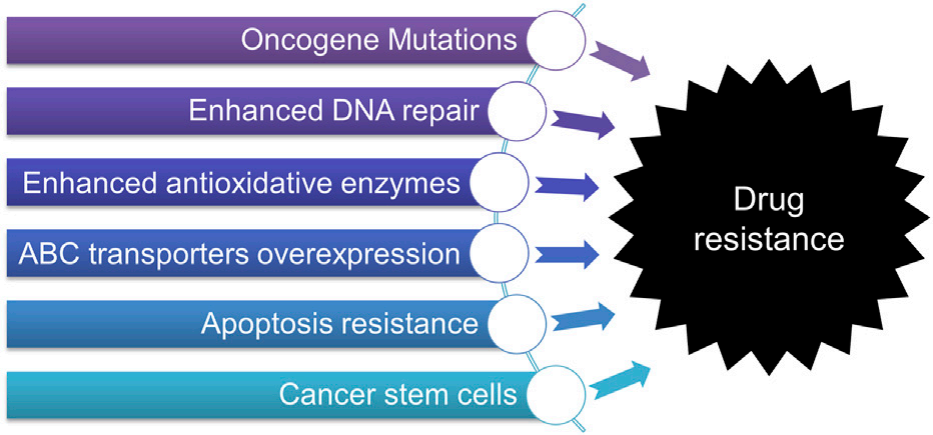
Multifaceted mechanisms contribute to drug resistance in cancers.

While variable and effective therapies are now available for most early- and certain late-stage cancers, a significant obstacle is the high incidence of innate or acquired drug resistance, which may eventually account for treatment failure and cancer-related death (Dong et al., 2022a; Cui et al., 2022; Wang et al., 2022). It has been confirmed that drug resistance can occur shortly following treatment of chemotherapies including conventional, targeted, and immunotherapy, posing a significant challenge in cancer treatment. Growing evidence has suggested that various factors contribute to drug resistance, such as 1) the mutations or alteration of oncogenes, 2) enhanced cellular defensive systems, including the activation of DNA repair, overexpression of ATP-binding cassette (ABC) transporters, and enhanced anti-oxidative activity, 3) apoptosis resistance, and 4) cancer stem cells (CSCs), *etc.* (Narayanan et al., 2020; Wang et al., 2021; Peery et al., 2022) as briefly discussed below and illustrated in Figure 1.

The mutations of oncogenes are the primary reasons that cause resistance to targeted therapies (Pagliarini et al., 2015). It has been reported that cancer cells can develop drug resistance after a short period of treatment by actively altering the associated genes not limited to oncogenes (Rosell, 2013). Specific mutations and alterations may confer universal resistance to conventional chemotherapeutics, targeted therapy, and cutting-edge immunotherapy (Zaretsky et al., 2016; Shin et al., 2017). A further structural modification or combinational strategy is usually adopted to overcome it.

The second direct way to escape from the toxic effects of anticancer agents is to activate cellular defensive weapons. Many conventional chemotherapeutics are known to induce DNA damage, thereby stopping cancer cell division and proliferation (Reuvers et al., 2020; Wettasinghe et al., 2021). However, cancer cells, especially resistant cells, are known to possess more robust phenotypes of antagonizing DNA damage via innate DNA repair or adaptive repair pathways (Jiang et al., 2020; Li et al., 2020). Another defensive weapon is the strengthened anti-oxidative activity via up-regulating reductive enzymes to reduce lethal levels of free radicals, which, in turn, help cancer cells evade cell death (Cui et al., 2018). Generally, due to unleashed cell proliferation, invasion, and migration, oxidative stress in cancer cells can be further increased due to drug exposure (Hayes et al., 2020). While most sensitive cells will be killed, a small fraction of surviving cells become resistant to the previously used drug by harnessing a more potent anti-oxidative mechanism (Okon and Zou, 2015). Combinational therapies are usually developed to overcome drug resistance mediated by enhanced DNA repair or antioxidative capability.

The overexpression of ABC transporters on cell membrane that can effectively transport anticancer drugs out of cancer cells is another leading cause of drug resistance (Li et al., 2016; Wu et al., 2022; Sajid et al., 2023). ABC transporters are a group of proteins composed of 49 members named ABCA-ABCG (Kathawala et al., 2015; Thomas and Tampe, 2020). Most of them have been validated in both lab and clinical studies to induce multi-drug resistance (MDR), a term describing cancer cells becoming resistant to a series of anticancer agents that are structurally and mechanistically distinct (Robey et al., 2018; Wang et al., 2021; Sajid et al., 2023). In recent two decades, while many specific or repurposed inhibitors/regulators of ABC transporters have been developed, their efficacies in clinical setting are yet to be validated (Wang et al., 2020; Dong et al., 2022b).

Depending on its mechanisms, most anticancer agents can induce apoptosis via external or internal pathways. However, cancer cells may swiftly upregulate anti-apoptotic but downregulate pro-apoptotic proteins, leading to drug resistance (Fulda, 2009; Neophytou et al., 2021). Novel agents targeting different players in apoptotic pathways are in urgent need.

CSCs are a set of sub-population cells with self-renewal and differentiation characteristics, possibly contributing to tumor formation and relapse (Shenouda et al., 2020). CSCs are naturally drug-resistant, possibly due to their intrinsic property and strengthened defensive weapons compared to non-CSCs (Carvalho et al., 2021; Fong et al., 2021; Miyoshi et al., 2021). By far, there are minimal therapies that can selectively target and eliminate CSCs.

Of note, the causes of resistance may be complicated and should be defined from case to case, those well-defined factors can also serve as feasible targets that can be modulated by pharmacological regulation, e.g., small-molecule agents.

### Bufalin derived from toad venom holds excellent promise in cancers

**FIGURE 2:**
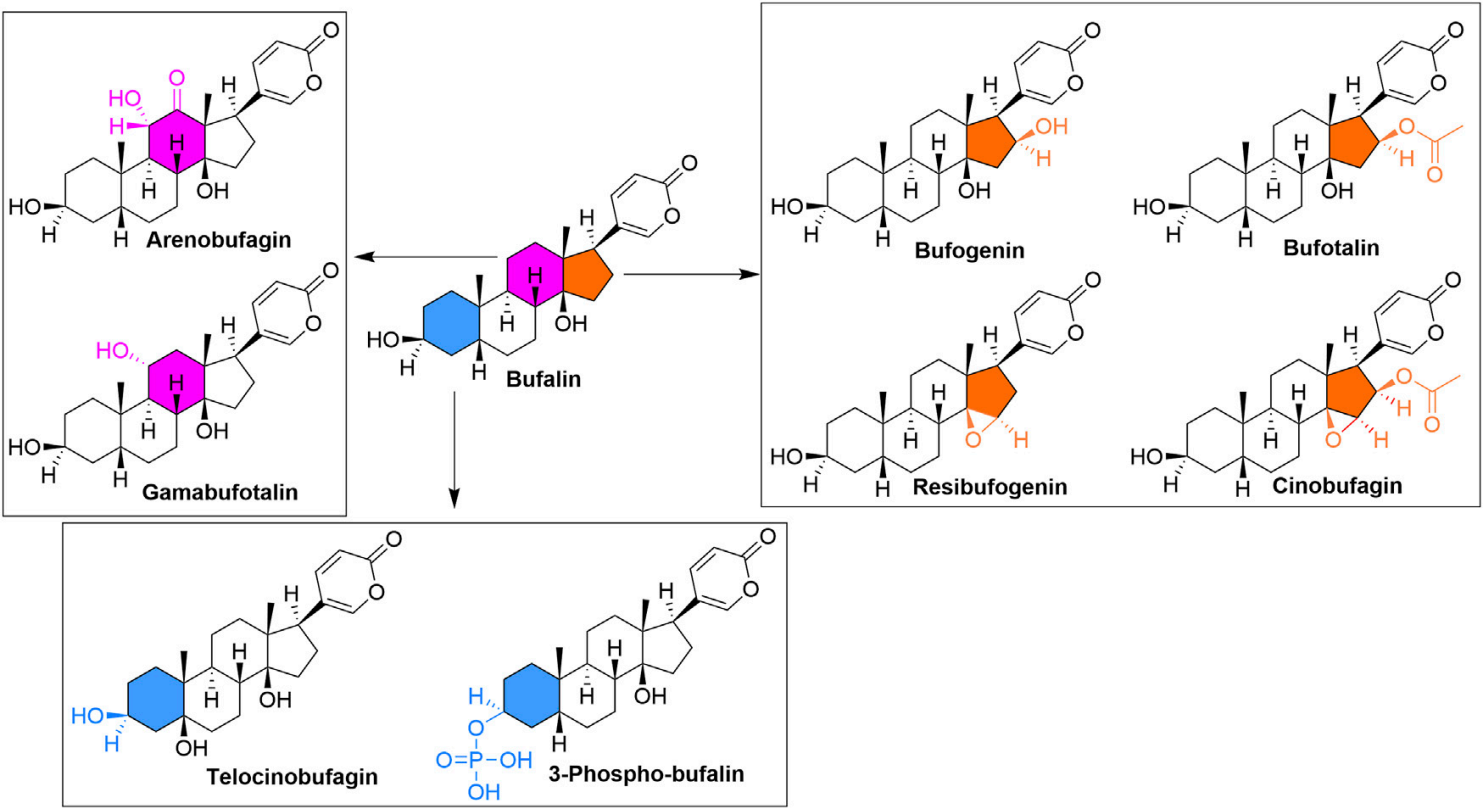
Structures of Bufalin and its derivatives. The primary structural differences in different rings are highlighted.

Toad venom (Chan-Su) is a traditional Chinese medicine (TCM) that has shown therapeutic efficacies for treating cancer, cardiovascular diseases, inflammation, *etc.* (Li et al., 2021; Zheng et al., 2022). Chemically, alkaloids (Dai et al., 2018a) and bufadienolides (Qu et al., 2012) are the two main components in toad venom that exert their pharmacological effects (Yang et al., 2015). Studies have confirmed that both alkaloids and bufadienolides majorly work to treat cancers (Zhang et al., 2013; Yu et al., 2014; Meng et al., 2016; Chen et al, 2020a; Dong et al., 2022a), while bufadienolides also exert therapeutic effects in cardiovascular diseases and inflammation (Zheng et al., 2022). Bufalin (Figure 2), 3β,14-dihydroxy-5β-bufa-20,22-dienolide (Zhang et al., 2020), has a molecular weight 386.53. Structurally, the other components of bufadienolides, including arenobufagin, gamabufotalin, bufogenin, bufatalin, resibufogenin, cinobufagin (Figure 2) which are all isolated or derived from toad venom, share the same scaffold as bufalin. Thus, these structurally related compounds can be regarded as bufalin’s derivatives. In this review, we would like to have an overview of bufalin, the most studied bufadienolide, in treating refractory and resistant cancers. Bufalin has been shown significant therapeutic effects in lung cancer (Zhu et al., 2012), bladder cancer (Hong and Choi, 2012), breast cancer (Yan et al., 2012a), oral cancer (Tsai et al., 2012), colon cancer (Xie et al., 2011), gastric cancer (Li et al., 2009), ovarian cancer (Takai et al., 2008), suggesting it is a broad-spectrum anticancer agent. Of note, bufalin has been extensively studied after 2012, especially in drug-resistant or refractory cancers. Therefore, in this review, we focused on those studies published in 2013–2022 (studies with significance will also be included).

Bufalin is historically recognized as a specific inhibitor of Na^+^/K^+^-ATPase, originally used to repel toad’s natural enemies (Laursen et al., 2015; Xu et al., 2016). While the major therapeutic application of bufalin falls in cancer treatment (Chen et al., 2021a), bufalin also possesses therapeutic effects on many other diseases, including inflammation and nociceptive pain (Rong et al., 2014; Wen et al., 2014; Zhakeer et al., 2017), trypanosomiasis (Rodriguez et al., 2020), which may indicate its versatile bioactivities that warrant further determination.

Bufalin was tested in a phase II clinical trial in China for pancreatic cancer (NCT00837239, initiated in 2009); however, no results were posted, and no other clinitrial trials were followed per ClinicalTrial.gov. By now, bufalin is mainly tested in the mainland of China.

### Bufalin exhibits excellent promise in refractory and drug-resistant cancers

#### Glioblastoma (GBM)

Malignant GBM is incurable and considered refractory cancer in the central nervous system because minimal therapeutic options are now available for adequate control (Simonds et al., 2021). Annually, more than 250,000 new cases are diagnosed, and there are over 200,000 deaths worldwide. The 5-year survival rate is only 7% for glioma (2019; Chen et al., 2021b). Due to the existence of the blood-brain barrier (BBB) that blocks the entrance of many exogenous chemical anticancer agents into the brain, GBM naturally possesses drug-resistance property (van Tellingen et al., 2015; Sarkaria et al., 2018). Bufalin may represent a promising therapy for GBM as it can penetrate BBB (Lan et al., 2018), thereby effectively 1) inducing apoptosis via a mitochondria-mediated pathway or 2) other different cell death ways such as necroptosis and 3) re-sensitizing specific chemotherapy.

A recent study by LingHu et al. (2020) showed that bufalin was effective in suppressing human glioma U-87 and U-373 cells, with IC_50_ value of ∼1 μM (MTT assay) in both cell lines after 24 h treatment (LingHu et al., 2020). Interestingly, they found that bufalin alone can trigger apoptosis, while when combined with zVAD.fmk (a caspase 8 inhibitor), it could switch to necroptosis, as supported by the correlative alteration of biomarkers of receptor-interacting serine/threonine-protein kinase 1 (RIPK1)/RIPK3, mixed lineage kinase domain-like protein (MLKL) and the formation of necrosome, all of which were necroptosis pathway-related proteins or markers. Further study indicated that the cytotoxicity of bufalin can be compromised by the silence of tumor necrosis factor-α (TNF-α) and TNF receptor 1 (TNFR1), whereas can be enhanced by the knockdown of both caspase 8 and Inhibitor of Apoptosis Proteins (IAP) cellular IAP1/2, suggesting its dual role in apoptosis and necroptosis (LingHu et al., 2020). This study indicated that apoptosis-resistant GBM may be sensitive to bufalin-induced necroptosis, warranting further investigation.

Lan et al. reported two studies using bufalin in treating GBM, and they revealed that sodium pump alpha1 subunit (ATP1A1) (Lan et al., 2018) and p53 (Lan et al., 2019) were two mediators through which bufalin exerted its anticancer activity. Bufalin is well-known to target ATP1A1 which also was proven to contribute to tumorigenesis (Wu et al., 2016; Feng et al., 2021). Lan et al. first validated that in U87MG, U251, and LN229 cells, the knockdown of ATP1A1 by siRNA suppressed the proliferation and colony formation. Similarly, bufalin (50 and 100 nM) downregulated ATP1A1, leading to inhibited cell proliferation which can be reversed by ATP1A1 siRNA treatment, providing a piece of indirect evidence that supported the on-target effect of bufalin toward ATP1A1 (Lan et al., 2018). The molecular biological study further revealed that bufalin did not alter ATP1A1 synthesis but could effectively induce ATP1A1 degradation via a proteasome-mediated mechanism. In the U87 xenograft model, bufalin reduced tumor growth in a dose-dependent manner (0.1, 0.5, 1, and 5 mg/kg), although it was toxic in reducing body weights of treated mice at 5 mg/kg. Hematoxylin and eosin (H&E) staining and immunohistochemistry (IHC) analysis of tumor tissues indicated that bufalin reduced the expression levels of both proliferating-cell nuclear antigen (PCNA) and ATP1A1, as well as S5a and PSMB5, two proteasome subunits, identical as observed in the cell-based assay (Lan et al., 2018). Later in 2019, the same group tested the therapeutic efficacies of bufalin in GBM U87, U251, LN229, A172, and U118 cells, and bufalin was found to possess IC_50_ values ranging from 50 to 120 nM (MTT assay) (Lan et al., 2019). Bufalin at 50 and 100 nM suppressed the colony formation and induced apoptosis of U87 and U251 cells via up-regulating pro-apoptotic proteins such as Bax, cleaved caspase 3/9, and cytochrome C, and down-regulating anti-apoptotic Bcl-2, all of which are well-known markers of the mitochondria-mediated apoptosis pathway. Bufalin appeared to induce DNA double-strand break (DSB) evidenced by up-regulating γ-H2AX, a marker of DNA damage, via translocation of p53 from the cytoplasm to nucleus mediated by down-regulating ATP1A1 and exportin 1 (XPO1) which functioned to export p53 from the nucleus to the cytoplasm. *In vivo* study showed that bufalin at 1 mg/kg significantly suppressed tumor growth of U87 (p53 wild type) but not U118 (p53 mutation) xenografts, with an inhibition rate of ∼60%. Additionally, the inhibition can be reversed by p53 inhibitor PIF (2 mg/kg), suggesting a p53-mediated mechanism (Lan et al., 2019).

Bufalin also appears to regulate particular microRNA since Liu et al. (2017) found that bufalin might suppress the proliferation and colony formation of U251 and U87 cells via regulating microRNA-203 (miR-203), whose over-express or downregulation resembled or antagonized bufalin’s effects *in vitro* (Liu et al., 2017). Bufalin increased miR-203 in U251 cells dose-dependently and time-dependently, which in turn down-regulating secreted protein acidic and rich in cysteine (SPARC), the target of miR-203, suggesting a network of bufalin, miR-203 and SPARC (Liu et al., 2017).

Shen et al. (2014) revealed the interaction of the cytotoxicity of bufalin with autophagy and endoplasmic reticulum (ER) stress (Shen et al., 2014). Bufalin suppressed U87MG glioma cells, with IC_50_ of 80–160 nM for a 24 h or 48 h treatment, respectively, resulting in cell apoptosis mediated by reactive oxygen species (ROS), upregulation of pro-apoptotic and downregulation of anti-apoptotic proteins (Shen et al., 2014). After bufalin treatment (20–80 nM), higher levels of ER stress sensors, including activating transcription factor 6 (ATF6), PKR-like ER kinase (PERK), eukaryotic translation initiation factor 2α (eIF2α), and inositol requiring enzyme 1 (IRE1) were identified. Furthermore, bufalin also increased C/EBP homologous protein (CHOP) level, whose knockdown could reverse bufalin’s effects (Shen et al., 2014). In addition to apoptosis, bufalin was also shown to induce autophagy, supported by the upregulation of LC3-II protein, an autophagy activation marker, which can be rescued by chloroquine, a lysosomotropic reagent that can inhibit autophagic flux. Bufalin was able to reduce ATP levels in U87MG cells in a time-dependent manner, accompanied by upregulated phosphorylation of AMP-activated protein kinase (AMPK) and downregulated phosphorylation of mammalian target of rapamycin (mTOR), which can be reversed by siRNA treatment targeting AMPK (Shen et al., 2014). Similar to the induction of apoptosis, ER stress, and PERK-eIF2α-CHOP axis played essential roles in bufalin’s effects on autophagy, which was attenuated by ER stress inhibitor tauroursodeoxycholate, while in contrast, enhanced when combined with autophagy inhibitor 3-methyladenine (Shen et al., 2014). The above information suggested that bufalin may induce varied types of cell death, including apoptosis, necroptosis, and autophagy.

Zhang et al. (2017) found that bufalin could work as an enhancer of radiotherapy in GBM U251 and U87MG cells. After a 48 h treatment, Bufalin had IC_50_ in U251 and U87MG at 250 nM and 150 nM, respectively, as determined by CCK8 assay (Zhang et al., 2017). Bufalin at 40 and 80 nM reduced EdU-positive cells in both cell lines, arresting cells majorly at the G2/M phase that led to the suppression of cell invasion and migration. Bufalin is known to induce apoptosis via a mitochondria-mediated mechanism. In this work, the authors further confirmed that bufalin (80 and 160 nM) dramatically reduced the oxygen consumption rate, which is a critical indicator represented mitochondrial respiratory function, leading to mitochondrial membrane potential (MMP) collapsing and reduced cellular ATP production (Zhang et al., 2017). Previously, Shen et al. (2014) found that bufalin may impair AMPK and mTOR pathways, which conferred ATP reduction (Shen et al., 2014); this study further validated that bufalin could target and disturb mitochondria directly. Radiation (4 Gy), when combined with bufalin (80 nM), showed improved effects in suppressing cancer cells and reducing colony formation via inducing DNA damage, as evidenced by the prolonged existence of γ-H2AX, probably mediated by impaired homologous recombination (HR) and associated RAD51, two DNA repair proteins (Zhang et al., 2017). This study provided experimental evidence using bufalin to sensitize or synergize with radiotherapy. Further validation is required in the animal study.

#### Triple-negative breast cancer (TNBC)

TNBC is defined to be human epidermal growth factor receptor 2 (HER2) negative and has <1% expression of estrogen receptors and progesterone receptors. TNBC, accounting for ∼15% of all breast cancer cases, has the poorest prognosis among all types of breast cancer, and there is no efficient targeted therapy but cytotoxic chemotherapy (Beebe et al., 2022; Li et al., 2022). Globally, there are more than two million newly diagnosed cases yearly (Giaquinto et al., 2022). While the 5-year survival rate for localized or regional is 91% and 65%, respectively, for distant TNBC is only 12% (Giaquinto et al., 2022). Since TNBC lacks specific oncogenes that can be targeted by modern precision medicine, effective agents are in urgent need.

Chen et al. (2020b) found that bufalin suppressed the proliferation (0.5 μM), colony formation (0.5 and 1 μM) of TNBC MDA-MB-231 and HCC-1937 cell lines, arresting cells at G2/M phase (Chen et al., 2020b), same as in GBM cells (Zhang et al., 2017). Bufalin (0.5 µM, 48 h) caused apoptosis of both cell lines at ∼10%. In the MDA-MB-231 xenograft model, bufalin (1 mg/kg, 3/week) showed a ∼60% inhibitory effect; however, no toxic effects were mentioned in this study. Bufalin inhibited the sphere formation of MDA-MB-231 and HCC1937 at 0.5 µM, accompanied by decreased levels of SOX2 and OCT4, two biomarkers of CSCs (Chen et al., 2020a).

Li et al. confirmed that the cell death induced by bufalin was not caspase-independent as pan-caspase inhibitor zVAD-fmk failed to rescue cell death in breast cancer MCF-7 and TNBC MDA-MB-231 cells (Li et al., 2018). Instead, necroptosis occurred following bufalin treatment, mediated by the upregulation of poly (ADP-ribose) polymerase-1 (PARP-1) and receptor-interacting protein (RIP)1/RIP3, especially in TNBC cells, which can be reversed by shRNA treatment targeting RIP3. Bufalin-induced ROS production (50 nM, 48 h) can also be reversed by a specific small molecule inhibitor of RIP1, necrostatin-1 (Nec-1, 20 μM), or N-acetyl-L-cysteine (NAC, 5 mM), an antioxidant agent. In the MAD-MB-231 cells xenograft model, bufalin (1 mg/kg, once/3 days) suppressed tumor growth, with an inhibitory rate of ∼60%, via inducing a clear necroptosis as shown in HE and TUNEL staining of tumor tissues. Meanwhile, these effects could be rescued by a PARP-1 inhibitor DPQ (5 mg/kg) co-treatment, suggesting a PARP-1 mediated pathway (Li et al., 2018). This study also showed that TNBC cells were more resistant to bufalin than other breast cancer cells.

Wang et al. (2016) found that miR-155-5p was upregulated after bufalin treatment in MDA-MB-231 cells and in adriamycin-resistant MCF-7/ADR cells (Wang et al., 2016). It appears that miR-155-5p plays a critical role in saving cancer cells since its overexpression could antagonize bufalin-induced apoptosis. In contrast, the downregulation of miR-155-5p could further sensitize apoptosis, suggesting the direct interaction of these two players. These effects seemed to be modulated via transcriptional factor forkhead box class O 3a (FOXO3a) and DNA methyltransferases 1 and 3a (DNMT1 and DNMT3a) (Wang et al., 2016). Intriguingly, the simultaneous inhibition of DNMT1 and DNMT3a also increased miR-155-5p expression, an effect resembling bufalin (Wang et al., 2016). More studies of miR-155-5p for its role in suppressing cancers are needed.

Steroid receptor coactivator 3 (SRC-3), one of three homologous members of the p160 SRC family, is believed to be bufalin’s target, which also positively correlated with poor prognosis of TNBC patients, serving as a marker for drug sensitivity and also prognosis (Tryfonopoulos et al., 2011; Wang et al., 2014; Kohale et al., 2022). Song et al. (2015) found that SRC-3 inhibitor bufalin effectively suppressed HCC1143, SUM149PT, SUM159PT, and MDA-MB-231 cells, with IC_50_ values ranging from 16 to 72 nM (MTT, 72 h). Bufalin (100 nM) downregulated SRC-3 in all the above cell lines, leading to repressed cell motility in MDA-MB-231-LM3-3 (Song et al., 2015). More importantly, bufalin (5 and 10 nM) could synergize with epidermal growth factor receptor (EGFR) inhibitor gefitinib, one of the tyrosine kinase inhibitors (TKIs), in LM3-3 cells. In this study, Song et al. synthesized a bufalin derivative to improve water solubility, 3-phosphate-bufalin (Figure 2), which showed higher blood concentration after intraperitoneal (IP) administration without showing any cardio-toxicity. In the orthotopic LM3-3 cells model, 3-phosphate-bufalin (0.75 mg/kg, 3/week) inhibited ∼50% of tumor growth without altering mouse body weight significantly, suggesting its effectiveness and safety. IHC assay also indicated the on-target effect of 3-phosphate-bufalin on SRC-3 (Song et al., 2015). This study provided another new chemical entity, 3-phosphate-bufalin, that has the potential to be evaluated further.

Recently, Liu et al. (2021) discovered that bufalin effectively inhibited MDA-MB-231, and two drug-resistant cell lines, including adriamycin-resistant MDA-MB-231/ADR cells and docetaxel-resistant MDA-MB-231/DOC cells via inducing both apoptosis at an early stage and necroptosis at a late stage, respectively (Liu et al., 2021). Bufalin inhibited all 3 cell lines in a concentration-dependent manner, with IC_50_ values of 304, 320, and 282 nM at 48 h by MTT assay, respectively, suggesting that these two resistant cancer cells did not show the cross-resistant property to bufalin (Liu et al., 2021). Hoechst33342/PI double staining showed after 24 h treatment of bufalin (300 nM), apoptosis was the primary form of cell death pattern in MDA-MB-231/ADR cells. In contrast, after 48 h, necroptosis was observed by transmission electron microscope (TEM), mediated by p-TNFR and p-RIP1. Interestingly, both necroptosis inhibitor Nec-1 (10–50 μM) and apoptosis inhibitor z-VAD-fmk (10–50 μM) could antagonize cell death induced by bufalin (300 nM), although Nec-1 to a greater extent. Their study also showed that the level of ROS increased significantly following bufalin treatment (300 nM, 48 h), leading to cell death that can be reversed by NAC or Nec-1, suggesting a RIP1-ROS axis in bufalin-induced cell death (Liu et al., 2021).

Two studies from Yan’s lab use bufalin to combine tumor necrosis factor-related apoptosis-inducing ligand (TRAIL) that is usually resistant to TNBC (Yan et al., 2012a; Yan et al., 2014). In these studies, bufalin was shown to possess IC_50_ values of 46.5 nM for MCF-7 and 513.3 nM for MDA-MB-231 cells, respectively, determined by MTT assay for a 48 h treatment. Bufalin induced apoptosis of these 2 cell lines, mediated by cleaved PARP (Yan et al., 2012a). TRAIL (100 ng/mL), when combined with bufalin (50 nM) showed enhanced activity in inducing apoptosis, increasing from 2% to 30% in MCF-7 cells and from 6.9% to 41% in MDA-MB-231 cells after 24 h treatment (Yan et al., 2012a; Yan et al., 2014), accompanied by the upregulation of death receptor 4 (DR4), DR5 and AMPK. At the same time, the knockdown of DR4/5 reduced apoptosis induced by this combination, suggesting the possible direct interaction between them. Further study showed that casitas B-lineage lymphoma-b (Cbl-b) but not Cbl-a might be the target through which bufalin sensitized TRAIL in inducing apoptosis. Moreover, the downregulation of Cbl-b by shRNA led to a more substantial synergistic effect of bufalin and TRAIL. This study suggested that the AMPK-DR4/5-Cbl-b loop played a central role in this combinational therapy, warranting further animal study of its efficacy.

The above studies suggested that TNBC cells may be more resistant to bufalin as compared to GBM cells since under most circumstances, bufalin possesses IC_50_s values in less than 200 nM in GBM, while in TNBC, it is in the ranges of 300–500 nM, suggesting the sensitivity of bufalin is cancer type (cells)-dependent.

#### Liver cancer

Liver cancer, also named hepatocellular carcinoma (HCC), ranks the third leading death among all cancer types after only lung and colorectal cancer. It was estimated that 905,700 people were diagnosed and 830,200 people died from liver cancer globally in 2020 (Ferlay et al., 2021). In 2040, it is predicted that 1.4 million people will be diagnosed with liver cancer (Rumgay et al., 2022). The 5-year relative survival rates for liver cancer are 35% (localized), 12% (regional), and 3% (distant) (Chen et al., 2021c). Bufalin shows great potential in treating liver cancer.

Fu et al. (2021) showed that bufalin might have effects in modulating immune systems in liver cancer cells. Bufalin (100–1,000 nM) dose-dependently inhibited the proliferation and induced apoptosis of human liver cancer SK-Hep1 and HepG2 cells (Fu et al., 2021). Bufalin pretreatment in SK-Hep1 cells (20 and 50 nM), HepG2 cells (50 and 100 nM), could significantly enhance the immune response mediated by natural killer (NK) cells via up-regulating the distribution on membrane but not the expression level of major histocompatibility complex class I-related chain A (MICA) on NK-92MI cells, mediated by down-regulating a disintegrin and metalloproteinase 9 (MMP9) which was shown to assist synthesized MICA secretion from cells (Fu et al., 2021). MICA was the primary ligand for the stimulatory receptor NKG2D on the surface of NK cells that regulated the immune response in NK cells to kill liver cancer cells. This study provided a novel combinational strategy to combat liver cancer (Fu et al., 2021).

Qiu et al. (2013) showed that bufalin at 10 and 100 nM effectively suppressed the proliferation, invasion, migration, and adhesion activity of HCCLM3 or HepG2 cells after 48 h treatment (Qiu et al., 2013). Bufalin (100 nM) time-dependently downregulated p-AKT and phosphorylated glycogen synthase kinase (GSK) protein but increased GSK3β protein activation and inhibited the translocation of β-catenin to nuclear, all of which work together to suppress liver cancer cells proliferation (Qiu et al., 2013). Further study showed that bufalin (100 nM) increases E-cadherin levels in HCCLM3 or HepG2 cells, decreasing matrix metalloproteinase-2 (MMP2) and MMP9 in HepG2 cells. Interestingly, bufalin increased MMP2 in HCCLM3, suggesting a cell type-dependent manner (Qiu et al., 2013). Further *in vivo* validation is warranted.

Recently, Ye et al. (2022) discovered a natural product, alisol B 23-acetate from *Alisma plantago-aquatica Linn* that was able to work synergistically with bufalin in liver cancer cells (Ye et al., 2022). Bufalin was able to inhibit the proliferation of SMMC-7721 and MHCC97 in the dose- and time-dependent manners, which can be further enhanced by the combination of alisol B 23-acetate, most likely through inducing apoptosis by mitochondria-mediated pathway as evidenced by upregulated Mcl-1, Bax, Bcl-2, and cleaved caspase-3 (Ye et al., 2022). In addition, this combination appeared to induce autophagy as supported by the increased level of LC3II/I mediated by Beclin-1 and p62. GSK-3β was found to be downregulated by the combination (Ye et al., 2022), a phenomenon similar to Qiu et al. (2013). A detailed analysis showed that Bufalin and alisol B 23-acetate could regulate the inactivation of the Wnt/β-catenin axis to suppress liver cancer. This study proposed an effective combination worth further testing in animal models.

As mentioned in the Introduction, bufalin is a toxic agent; thus, its role as a chemo-sensitizer other than an anticancer agent at low doses is worth trying. Four studies showed bufalin may have synergistic effects when combined with sorafenib, a TKI that targets vascular endothelial growth factor receptor (VEGFR) (Gao et al., 2012; Zhai et al., 2015; Wang et al., 2018a; Wang et al., 2018b).

Bufalin (25–200 nM) worked synergistically with sorafenib (2.5–10 μM) to reduce the growth of HepG2 and Huh7 cells via inducing apoptosis, as shown in Zhai et al. (2015). Bufalin (100 nM) appeared to overcome sorafenib resistance via targeting p-Akt, which can be further enhanced by either co-treatment of Akt inhibitor perifosine or the depletion of Akt. Akt inactivation induced by bufalin was shown to be IRE1-dependent, while independent of eIF2 or CHOP. Additionally, they showed that bufalin reversed sorafenib resistance in sorafenib-resistant Huh7-Sora cells via down-regulating p-Akt, which can be reversed by siRNA targeting IRE1 (Zhai et al., 2015).

In PLC/PRF/5 and SMMC7721 cells, bufalin (20 nM) and its combination with sorafenib (10 μM) exerted the most potent suppressing effects than other tested combinations, possible via inducing mitochondria-mediated apoptosis as evidenced by increased Bax, PARP, and caspase 7 (Wang et al., 2018a). This combination did not alter the cell cycle (Wang et al., 2018a). Another study revealed that a combination of sorafenib (6.25 μM) and bufalin (50 nM), with a fixed ratio of 25:1 showed the most potent apoptosis-inducing effects in PLC/PRF/5 and HepG2 cells (Gao et al., 2012). In addition to the inhibition of p-Akt, this combination appeared to suppress p-ERK, and a PI3K inhibitor LY294002 could reverse the p-ERK downregulation due to the combination of bufalin and sorafenib (Gao et al., 2012). Wang et al. (2018b) further validated the *in vivo* antitumor effects of combining bufalin with sorafenib (Wang et al., 2018b). In the SMMC-7721 cells xenograft model, bufalin (1 mg/kg, 5/week, IP), when combined with sorafenib (30 mg/kg/day, 5/week, oral) showed better tumor-reducing effects as compared to mono-therapy. p-AKT, VEGF, and mTOR but not p-ERK in tumor tissues were found to be downregulated (Wang et al., 2018b), suggesting that this combination might work differently *in vivo* as compared to *in vitro* (in which p-ERK was also downregulated by bufalin and sorafenib).

MDR poses a significant threat to effective cancer therapies. One of the leading causes of MDR is the overexpression of specific ABC transporters such as P-glycoprotein (P-gp) and multi-drug resistance protein 1 (MRP1) (Narayanan et al., 2021; Wang et al., 2021). Gu et al. (2014) found that bufalin might overcome MRP1-mediated 5-fluorouracil (5-FU) resistance in 5-FU-resistant liver cancer BEL-7402/5-FU cells (Gu et al., 2014). Bufalin was highly effective in arresting cells at the G0/G1 phase and inhibiting BEL-7402/5-FU cells by inducing apoptosis, with an IC_50_ value of 80 nM. Bufalin at a non-toxic concentration of 1 nM could significantly enhance the sensitivity of 5-FU in these resistant cells. The mechanistic study showed that bufalin was able to inhibit MRP1’s efflux, as it can increase the cellular concentration of Rhodamine-123, adriamycin, and 5-FU, all of which were the substrates of MRP1, probably through down-regulating the mRNA and expression level of MRP1 (Gu et al., 2014). This study may indicate that bufalin has the potential to circumvent MDR that is mediated by MRP1, and possibly other ABC transporters as well. Since these transporters confer resistance to not only conventional chemotherapy but also many targeted therapies, thus, it is worth trying for bufalin’s complete applications via combination.

Xu et al. (2021) constructed albumin nanoparticles that could deliver bufalin and TKI nintedanib for liver cancer treatment (Xu et al., 2021). These well-prepared nanoparticles, BF-ND-BUP-sMPs, showed stable releasing of both bufalin and nintedanib. They exhibited good biocompatibility with HepG2 cells, and much decreased IC_50_ compared to bufalin or nintedanib alone. BF-ND-BUP-sMPs, at an equivalent dose of 0.8 mg/kg of bufalin and 1.05 mg/kg of nintedanib, showed the most potent effect in suppressing tumor growth of H22 cells xenograft model, with an inhibitory rate of over 80%, without showing any weight loss or any significant damage to heart, liver, spleen, lung, and kidney (Xu et al., 2021). This study suggested that the constructed nanoparticles were safe, effective, and stable, warranting further evaluations.

#### Pancreatic cancer

As one of the most aggressive human malignancies, pancreatic cancer is a leading cause of cancer-related deaths worldwide. Only 12% of patients will live 5 years after diagnosis (all stages combined) (Rawla et al., 2019). The majority of patients are diagnosed with an unresectable or metastatic disease that has inferior prognosis and low survival rate (Bengtsson et al., 2020). Bufalin has demonstrated strong potency in pancreatic cancer by targeting c-Myc and several other players.

Liu et al. (2016) reported that in the BxPC3-luc2 xenograft model, bufalin at 1 or 2 mg/kg was able to reduce tumor growth dramatically (∼50% reduction) without showing any weight loss (Liu et al., 2016). Interestingly, in this study, 2 mg/kg of cisplatin was toxic in reducing body weight. Bufalin highly effectively (starting from as low as 10 nM) suppressed the proliferation of human pancreatic cancer Sw1990 and BxPc3 cells through inducing cell cycle arrest at the S phase mediated by down-regulating c-Myc and NF-κB expression as determined by luciferase assay and Western blot (Liu et al., 2016). Thus, in addition to other reported targets, bufalin inhibits c-Myc, requiring further validation.

Tian et al. (2015) identified another potential target of bufalin, human telomerase reverse transcriptase (hTERT), which partially protected mitochondria from damage due to ROS (Tian et al., 2015). In this study, bufalin possesses an IC_50_ of 159.2 nM in CAPAN-2 human pancreatic cancer cells. Bufalin (50, 100, and 150 nM) decreased the level of hTERT but increased the levels of p-JNK and p-p38, suggesting it may exert its effects partially via JNK/p38 pathway since the blockage of this pathway (by JNK inhibitor SP600125 or p38-MAPK inhibitor SB203580) could rescue cell death induced by bufalin. In addition, the silence of hTERT showed similar cancer-suppressing effects as bufalin (Tian et al., 2015). This study and the above information suggested that bufalin is a multi-targeting compound.

Bufalin appears to enhance the sensitivity of gemcitabine in pancreatic cancers (Chen et al., 2012; Li et al., 2014; Wang et al., 2016). Bufalin inhibited PANC-1 and CFPAC-1 cells via arresting cell cycle at G2/M and inducing apoptosis mediated by down-regulating p-Akt and anti-apoptotic protein heat shock protein 27 (Hsp27) (Li et al., 2014). Combining 50–100 nM bufalin with 500 nM gemcitabine in PANC-1 and CFPAC-1 showed more potent cell-suppressing effects than either drug alone (Li et al., 2014). Bufalin (10 nM), when combined with gemcitabine (0.5–1.5 μg/mL), showed higher suppressive effects in pancreatic cancer Bxpc-3, MiaPaCa-2, and PANC-1 cells via inducing apoptosis mediated by cleaved caspase 3, Bcl-2, apoptosis signal-regulating kinase 1 (ASK1)/JNK (Chen et al., 2012). In the MiaPaCa-2 xenograft model, the combination of bufalin (0.1 mg/kg) with gemcitabine (125 mg/kg) showed the most potent tumor-suppressing effect among other single-use. Furthermore, the IHC examination also validated the upregulation of ASK1 due to the combination (Chen et al., 2012).

In another study in gemcitabine-resistant human pancreatic cancer cell line MiaPaCa2/GEM that has a significant fraction of CSCs, bufalin (50 nM) effectively suppressed the proliferation and sphere formation, accompanied by downregulated CD24 and epithelial specific antigen (ESA), two markers of pancreatic CSCs (Wang et al., 2016). While bufalin (1.5 mg/kg, 5 days/week) did not reduce the tumor volumes of the MiaPaCa2/GEM xenograft model, it reduced tumor weight significantly as compared to the untreated group, without showing a noticeable toxic effect (Wang et al., 2016). Bufalin also inhibited metastasis of MiaPaCa2/GEM cells in the animal model, mediated probably by the Hedgehog signaling pathway as supported by downregulated levels of PTCH2 and Gli1 (Wang et al., 2016). Unfortunately, this study did not determine the combined effect of bufalin with gemcitabine.

#### Lung cancer

Lung cancer ranks first in the deaths caused by cancers (Sung et al., 2021). While the prognosis may be good if diagnosed early, drug resistance is still a significant obstacle (Tumbrink et al., 2021; Ashrafi et al., 2022; Su, 2022). Bufalin showed great promise in treating drug-resistant lung cancers.

Two studies showed that bufalin could work with gefitinib, a TKI targeting EGFR. Huang et al. (2016) showed that bufalin (0–60 nM, 48 h) decreased cell viability, adhesion, and mobility of gefitinib-resistant NCI-H460/G lung cancer cells, resulting in suppressed cell invasion and migration (Huang et al., 2016). It was shown that bufalin (2.5–10 nM) decreased SOS-1, MMP2, and RhoA, three essential metastasis-related proteins, but increased p38, urokinase plasminogen activator (uPA), p-focal adhesion kinase (FAK), p-ERK1/2, Ras, E-cadherin and tissue inhibitor matrix metalloproteinase 1 (TIMP1) (Huang et al., 2016). However, no *in vivo* anticancer confirmation has been revealed.

Met-Hepatocyte growth factor (HGF) axis is essential in tumor progression and drug sensitivity (Huang et al., 2020; Fu et al., 2021). In EGFR mutant human lung adenocarcinoma cell lines PC-9, HCC827, and H1975 cells, exogenous HGF conferred resistance to gefitinib, which can be reversed by bufalin (20 nM) co-treatment via down-regulating Met/PI3K/Akt signaling (Kang et al., 2013). Bufalin restored the sensitivity of gefitinib in the presence of HGF by inducing apoptosis mediated by cleaved-PARP cleaved-caspase-3/9 (Kang et al., 2013).

In addition, bufalin was found to exert similar effects towards another EGFR TKI afatinib in afatinib-resistant H1975 lung cancer cells (H1975AR) via the exact mechanism as above, except bufalin’s effect in increasing E-cadherin (Kang et al., 2015). However, no *in vivo* study was ever conducted.

Another similarity to bufalin in liver cancer is that the combination with bufalin in lung cancer could also sensitize sorafenib (Kuo et al., 2022). Kuo et al. (2022) recently showed that bufalin (60, 90, and 120 nM) could significantly enhance the activity of sorafenib (10, 15, and 20 μM) in reducing the cell viability of human lung cancer NCI-H292 cells via inducing apoptosis through ROS (Kuo et al., 2022). Lower anti-apoptotic Bcl-2 expression but higher pro-apoptotic proteins Bax, Bad, APAF-1, and caspase-3/9 were identified after the combination treatment, suggesting a mitochondria-mediated mechanism (Kuo et al., 2022).

Bufalin also appears to augment adriamycin’s activity in A549 cells, as shown in Zhang et al.’s study (2017). Combining bufalin (1, 20, 100 nM) with adriamycin (1 μM) significantly increased the growth inhibition rate of A549 cells in a time-dependent manner than either monotherapy. This combination induced apoptosis mediated by increased caspase-3 and cell cycle arrest at the S phase (Zhang and Fu, 2017). No animal model was used to validate the efficacy of bufalin combined with adriamycin.

Clearly, more animal experiments are required to validate bufalin and its sensitization effects with other anticancer agents.

#### Colorectal cancer (CRC)

CRC has a poor prognosis because a significant proportion of cases are diagnosed at later stage, with very few effective treatments available (Siegel et al., 2020). In addition, drug resistance is another major challenge, in which bufalin shows promising therapeutic results (Hu et al., 2016; Chen et al., 2022).

Hypoxia is a critical player in inducing drug resistance, including photodynamic therapy (PDT). Recently, Yuan et al. (2022) constructed nanoparticles composed of bufalin and a PDT mTHPC, which was named T-B@NP that has been shown to stably release bufalin, thereby inhibiting hypoxia inducible factor 1α (HIF-1α) and overcoming HIF-1α-mediated resistance to mTHPC in HCT116 and CT26 cells (Yuan et al., 2022). T-B@NP preferably targeted and accumulated in tumor tissues, leading to more potent tumor-inhibiting effects (∼90%) when combined with laser treatment among all other single therapies, without reducing body weight (Yuan et al., 2022). This study provided a combinational strategy of bufalin with PDT, and the developed nanoparticles T-B@NP are worthy of further evaluation.

Dai et al. (2018b) found that bufalin (10, 20, and 30 nM) enhanced the cell-suppressing effect of 5-FU (5, 10, and 15 μM) in dose- and time-dependent manners, with a combination index (CI) < 1 which suggested strong synergistic effects (Dai et al., 2018b). Among all tested concentrations, 30 nM bufalin combined with 15 μM 5-FU showed the most vigorous activity as determined by CI (∼0.55). This optimized combination therapy showed superior activity in inducing apoptosis via up-regulating the expression levels of cleaved caspase-3/9, Bax, Bad, and cleaved PARP, meanwhile down-regulating Bcl-2, IAPs XIAP, and survivin, *etc.* Further study suggested that Bax was required for apoptosis induced by this combination since the silence of Bax by siRNA reversed apoptosis (Dai et al., 2018a). *In vivo* study is needed to validate the efficacy and safety.

Like in other cancer types, bufalin can also suppress colorectal CSC. Sun et al. (2017) reported that a low dose of cisplatin (≤5 μM) might enhance the stemness of human CRC HCT116 and LoVo cells as determined by tumorsphere formation (Sun et al., 2017). Cisplatin could further augment the stemness of cisplatin-pretreated CRC cells, which can be antagonized by bufalin at 1 nM as shown by tumorsphere formation assay, side-population (SP) cells analysis, and Hoechst 33342 staining assay (Sun et al., 2017). Bufalin reduced the levels of CD133, CD44, NANOG, OCT4, SOX2, and ABCG2, six markers of CSCs induced by cisplatin, thereby enhancing the sensitivity of cisplatin in cisplatin-resistant CRC cells. In the HCT116 xenograft model, bufalin (1 mg/kg/3 days) significantly sensitizes cisplatin (10 mg/kg) in reducing tumor weight (∼60% reduction), accompanied by decreased levels of all six CSC markers in tumor tissues (Sun et al., 2017).

#### Prostate cancer

Prostate cancer is a severe disease that undermines men’s health since it is the second most prevalent among men and the second leading cause of death in men (Rawla, 2019). The vast majority of prostate cancer patients will eventually develop resistance to androgen deprivation therapy (ADT) (Petrylak, 2013; Nakazawa et al., 2017). Bufalin effectively suppresses prostate cancer DU145 and PC-3 cells via regulating p53 or particular miRNA (Zhang et al., 2017; Zhang et al., 2017), and can work synergistically with other anticancer agents.

Since bufalin itself is a highly toxic agent, thus, Gu and Zhang. (2021) explored the combination of DNA topoisomerase I (Top1) inhibitor hydroxycamptothecin with low-dose bufalin (Gu and Zhang, 2021). In this study, castration-resistant prostate cancer (CRPC) DU145 cells xenograft model in nude mice were constructed and treated by hydroxycamptothecin (2 mg/kg) combined with bufalin at 0.4, or 0.6, or 0.8 mg/kg, respectively. Among the three tested regimens, 0.6 mg/kg bufalin, when combined with hydroxycamptothecin, named as H6B, showed the most potent tumor-reducing effect, with an inhibitory rate of roughly 80% without showing noticeable toxic effect to reduce body weight (Gu and Zhang, 2021). H6B efficiently suppressed cancer cell proliferation via inducing apoptosis through a mitochondria-mediated mechanism since it increased pro-apoptotic Bax, p53, and PDCD4. In contrast, it decreased anti-apoptotic Bcl-XL and p-Akt as per Western blot assay (Gu and Zhang, 2021). This safe and effective regimen suggests a broader screening of the combination of bufalin with other FDA-approved Top1 inhibitors. In addition, further evaluation of H6B in more animal models and possibly in humans is warranted.

#### Cervical cancer

Cervical cancer is one of the leading gynecological malignancies worldwide and is often diagnosed at an advanced stage that lacks effective therapy. The 5-year survival rate of cervical cancer patients with stage III was 32.8%, but those with stage IV were only 7.1% (Zhou et al., 2020; Choi et al., 2021). Bufalin may work efficiently with paclitaxel in cervical cancer as shown in Liu et al. (2016). Bufalin dose-dependently inhibited the proliferation and colony formation of cervical cancer Siha (IC_50_ ∼160 nM) and HeLa (IC_50_ ∼80 nM by CCK-8) cells via 1) arresting cell cycle at G2/M phase mediated by down-regulating cyclinA/CDK2 and 2) inducing apoptosis mediated by up-regulating Bax and down-regulating Bcl-2 and Bcl-xL (Liu et al., 2016). Furthermore, bufalin (20 and 40 nM) suppressed the invasion and migration of Siha and Hela cells. The mechanistic study indicated that bufalin (20 nM *in vitro* and 10 mg/kg/4 days *in vivo*) impacted the integrin α2/β5/FAK signal pathway, leading to enhanced cytotoxicity of paclitaxel (5 nM *in vitro* and 10 mg/kg/4 days *in vivo*) in cells and in Siha xenograft model, without demonstrating the obvious toxic effect (Liu et al., 2016). IHC staining in xenograft tumor tissues confirmed the on-target effect of bufalin on integrin α2, integrin β4, and FAK (Liu et al., 2016).

#### Osteosarcoma

Osteosarcoma is the second leading cause of cancer-related death in children and young adults (Misaghi et al., 2018; Bielack et al., 2022). Chang et al. (2015) successfully isolated CSCs from primary osteosarcoma cells derived from a patient’s tumor tissue, which were named C1OS-CSCs, and they evaluated the inhibiting potential of bufalin in these cells (Chang et al., 2015). Bufalin (10 µM) decreased sphere formation via down-regulating ALDH1, TERT, NANOG, CD133, Notch, and Bim1, all of which were biomarkers of CSCs. When injected in nude mice, the pretreated C1OS-CSCs showed reduced activity in forming a tumor, suggesting the reduced stemness, likely due to bufalin pretreatment. The authors screened the changes in miRNA levels, and identified miR-148a as a potential target of bufalin, which downregulated DNMT1 and p27 to modulate the stemness of C1OS-CSCs (Chang et al., 2015). Further *in vivo* evaluation is necessary to assess bufalin for osteosarcoma. Notably, the high concentration they used in this study, 10 μM, was about 100–1,000 folds higher than those conducted in other studies.

Bufalin also shows potential in treating drug-resistant bladder cancer, gastric cancer, multiple myeloma, and leukemia.

#### Bladder cancer

Bufalin (5 and 10 nM) showed synergistic effects with TRAIL (25 and 50 ng/mL) in suppressing the proliferation of human bladder carcinoma T24 cells via inducing apoptosis mediated by up-regulating DR4 but down-regulating DR5 (Kang et al., 2017). Further study showed that these effects may also involve XIAP, Bid, and cFLIP, as well as caspases 3/8/9, through which the details remain to be revealed (Kang et al., 2017). No animal model was used to confirm bufalin efficacy.

#### Gastric cancer

It has been shown that the Akt pathway activation can confer cisplatin resistance in gastric cancer, as shown in the study by Zhao et al. (2016) (Zhao et al., 2016). Bufalin (50, 100, and 200 nM) downregulated the level of p-Akt but not the overall level of Akt, leading to a synergistic effect in inhibiting cell proliferation and inducing apoptosis of human gastric cancer SGC7901, MKN-45, and BGC823 cells. The mechanistic study in SGC7901 cells showed that bufalin downregulated p-Akt and its downstream p-GSK3β, p-mTOR, P-4EBP1, and p-S6K, which may work together to enhance the cytotoxicity of cisplatin (Zhao et al., 2016).

#### Multiple myeloma

MK2206 is an Akt inhibitor under multiple clinical trials (Oki et al., 2015; Xing et al., 2019; Chien et al., 2020). As discussed above, bufalin can suppress the activation of Akt; thus, it is reasonable to assume that these two agents may work together. Huang et al. (2018) found that bufalin had an IC_50_ value of 10–20 nM (48 h) in multiple myeloma cell line H929 (Huang et al., 2018). Bufalin (20 nM), when combined with MK2206, showed a higher inhibiting rate of cell proliferation than mono-therapy of either agent via inducing apoptosis mediated by cleaved caspase 3 and cleaved PARP (Huang et al., 2018). Interestingly, bufalin alone could increase p-Akt, which MK2206 can reverse. Bufalin’s effect in increasing p-Akt apparently contradicted with the above information. Thus, further mechanistic and *in vivo* studies are needed to confirm the anticancer potential.

#### Leukemia

Bufalin also exerted potential therapeutic application in leukemia as it, at non-toxic doses, was proven to activate immune responses in the WEHI-3 cell-generated leukemia *in vivo* model (Shih et al., 2018). However, bufalin’s potential in leukemia remains to be exploited.

## Discussion

### Summary of bufalin in refractory and drug-resistant cancers

**FIGURE 3:**
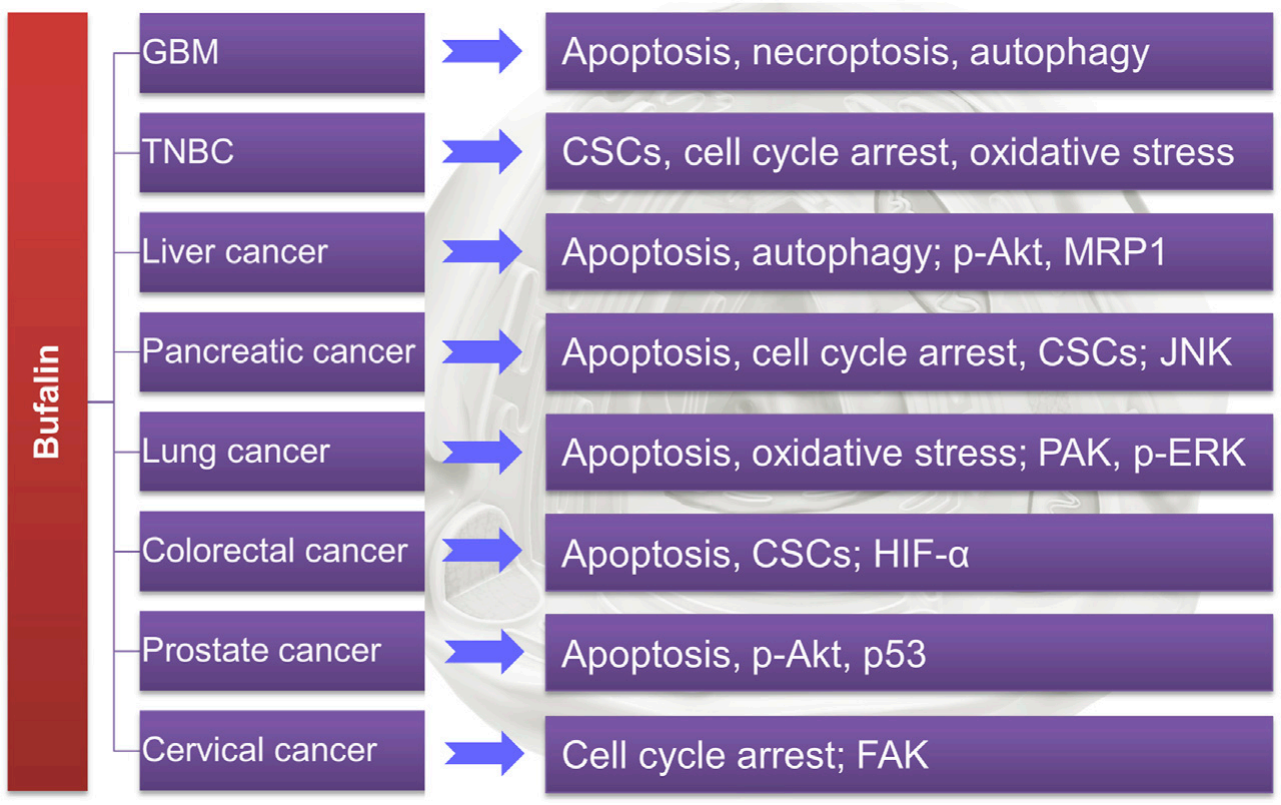
Bufalin has shown potential in treating refractory cancers by regulating various targets.

The above review has summarized the application of TDAAs in PCa (as also shown in Table 1). It is known that bufalin targets SRC-1/3 for cancer treatment (Wang et al., 2014), while growing evidence suggests that bufalin could also target or downregulate many essential enzymes/proteins in cancer cells, including ATP1A1 (Lan et al., 2018), specific miRNA (Liu et al., 2017), AMPK/mTOR pathway (Shen et al., 2014), p-Akt (Wang et al., 2018b) and Met/PI3K/Akt pathway (Kang et al., 2013), MRP1 (Gu et al., 2014) *etc.*, suggesting that it is a multi-targeting or multi-functional agent. Generally, bufalin could suppress refractory and drug-resistant cancer cells *in vitro* and *in vivo* via inducing DNA damage, apoptosis, necroptosis, autophagy, oxidative stress, and cell cycle arrest, *etc.*, as summarized in Table 1 and Figure 3.

**TABLE 1:** Summary of bufalin in refractory or drug-resistant cancers.

Cancer type	Mechanisms/Targets	Effects	Refs
GBM	Inducing apoptosis and necroptosis mediated by RIPK1/3, MLKL, TNF-α, TNFR1	Suppress U-87 and U-373 cells	LingHu et al. (2020)
	Targeting ATP1A1, p53	Suppress U87MG, U251 and LN229 cells	Lan et al. (2018)
	DNA damage, mitochondria-mediated apoptosis, p53 miR203, SPARC	Inhibit U87MG xenograft tumors	Lan et al. (2019)
	Autophagy, ER stress, AMPK/mTOR, PERK-eIF2α-CHOP axis	Suppress U87, U251, LN229, A172, and U118 cells	Liu et al. (2017)
	AMPK/mTOR, cell cycle arrest at G2/M	Inhibit U87 xenograft tumors Suppress U87 and U251 cells Suppress U87MG cells Synergism with radiation in U251 and U87MG cells	Shen et al. (2014), Zhang et al. (2017)
TNBC	CSCs, cell cycle arrest at G2/M	Suppress MDA-MB-231 and HCC-1937 cells	Chen et al. (2020a)
	Necroptosis mediated by PARP-1, RIP1/3, oxidative stress	Inhibit MDA-MB-231 xenograft tumors	Li et al. (2018)
	miR-155-5p, FOXO3a, DNMT1/3a	Suppress MDA-MB-231 cells	Wang et al. (2016)
	SRC-3	Inhibiting MDA-MB-231 xenograft tumors	Song et al. (2015)
	Inducing apoptosis and necroptosis mediated by RIP1 and ROS	Suppress MDA-MB-231 cells and adriamycin-resistant MCF-7/ADR cells	Liu et al. (2021)
	DR4/5, AMPK, Cbl-b	Suppress HCC1143, SUM149PT, SUM159PT and MDA-MB-231 cells	Yan et al. (2012a), Yan et al. (2014)
		Synergism with gefitinib in LM3-3 xenograft	
		Suppress MDA-MB-231, adriamycin-resistant MDA-MB-231/ADR cells and docetaxel-resistant MDA-MB-231/DOC cells	
		Synergism with TRAIL in MDA-MB-231 cells	
		Suppress MCF7 and MDA-MB-231 cells	
Liver cancer	Inducing apoptosis and modulating immune systems	Suppress SK-Hep1 and HepG2 cells	Fu et al. (2021)
	Down-regulating p-Akt, GSK, MMP2/9	Suppressing HCCLM3 and HepG2 cells	Qiu et al. (2013)
	Inducing apoptosis and autophagy p-Akt	Synergism with alisol B 23-acetate in SMMC-7721 and MHCC97 cells	Ye et al. (2022)
	Inducing mitochondria-mediated apoptosis p-Akt, p-ERK	Synergism with sorafenib in HepG2 and Huh7 cells	Zhai et al. (2015)
	p-AKT, VEGF and mTOR	Synergism with sorafenib in PLC/PRF/5 and SMMC7721 cells	Wang et al. (2018a)
	MRP1	Synergism with sorafenib in PLC/PRF/5 and HepG2 cells	Gao et al. (2012)
	Undefined	Synergism with sorafenib in SMMC-7721 cells xenograft model	Wang et al. (2018b)
		Reverse 5-FU resistance in BEL-7402/5-FU cells	Gu et al. (2014)
		Synergism nintedanib in H22 cells xenograft model	Xu et al. (2021)
Pancreatic cancer	c-Myc, cell cycle arrest at S	Inhibit BxPC3-luc2 xenograft tumors	Liu et al. (2016)
	hTERT, JNK/p38	Suppress CAPAN-2 cells	Tian et al. (2015)
	Inducing apoptosis and cell cycle arrest at G2/M	Synergism with gemcitabine in PANC-1 and CFPAC-1 cells	Li et al. (2014)
	Inducing apoptosis, SAK1/JNK	Suppress Bxpc-3, MiaPaCa-2 and PANC-1 cells	Chen et al. (2012)
	CSCs	Synergism with gemcitabine in MiaPaCa-2 xenograft model	Wang et al. (2016)
		Inhibiting MiaPaCa2/GEM xenograft tumors	
Lung cancer	p38, FAK, p-ERK, E-cadherin	Suppress proliferation, invasion and migration of gefitinib-resistant NCI-H460/G cells	Huang et al. (2016)
	Met/PI3K/Akt	Synergism with gefitinib in PC-9, HCC827, and H1975 cells	Kang et al. (2013)
	ROS, apoptosis	Synergism with sorafenib in NCI-H292 cells	Kuo et al. (2022)
	Apoptosis and cell cycle arrest at S	Synergism with adriamycin in A549 cells	Zhang and Fu (2017)
Colorectal cancer	HIF-1α	Synergism with mTHPC in HCT116 and CT26 cells	Yuan et al. (2022)
	Inducing mitochondria-mediated apoptosis	Synergism with mTHPC in CT26 cells xenograft model	Dai et al. (2018a)
	CSCs	Synergism with 5-FU in HCT116	Sun et al. (2017)
		Synergism with cisplatin in cisplatin-resistant HCT116 cells	
Prostate cancer	Apoptosis, p-Akt, p53	Synergism with hydroxycamptothecin in DU145 cells xenograft model	Gu and Zhang (2021)
Cervical cancer	Cell cycle arrest at G2/M, integrin α2/β5/FAK	Synergism with paclitaxel *in vitro* and in Siha xenograft model	Liu et al. (2016)
Osteosarcoma	CSCs	Suppress C1OS-CSCs cells	Chang et al. (2015)
Bladder cancer	Apoptosis mediated by DR4/5	Synergism with TRAIL in T24 cells	Kang et al. (2017)
Gastric cancer	p-Akt	Synergism with cisplatin in SGC7901, MKN-45 and BGC823 cells	Zhao et al. (2016)
Multiple myeloma	p-Akt, apoptosis	Synergism with MK2206 in H929 cells	Huang et al. (2018)
Leukemia	Immune response	Undetermined	Shih et al. (2018)

The authors would like to discuss several characteristics of bufalin.

### Bufalin is effective in inducing apoptosis, necroptosis, and autophagy

Apoptosis, formerly known as programmed cell death, can be initiated via either an external or internal pathway. Bufalin has been validated to induce internal apoptosis, primarily mediated by mitochondria and other central players, including cleaved caspases, cleaved PARP, and pro-apoptotic proteins (Lan et al., 2019; LingHu et al., 2020). This commonly shared mechanism by other well-known anticancer agents does not make bufalin an outlier. Additionally, at the late stage of bufalin treatment, e.g., after 48 h, it can activate necroptosis, an alternative mode of regulated cell death mimicking features of apoptosis and necrosis (LingHu et al., 2020). Necroptosis requires the protein RIPK3 (previously well-recognized as a regulator of inflammation, cell survival, and disease) and its substrate MLKL, the crucial players of this pathway (Morgan and Kim, 2022). It has been shown that bufalin may target both RIPK3 and MLKL, thereby leading to necroptosis and the subsequent proliferation inhibition of GBM and TNBC cells (Li et al., 2018; LingHu et al., 2020).

Autophagy is a cytoprotective biological event which also serves as a vulnerability in cancer cells (Yu et al., 2018; Mulcahy and Thorburn, 2020). Interestingly, specific anticancer agents can either activate or inhibit autophagy (Li et al., 2017). For bufalin, autophagy is activated, as evidenced by LC3-II, the central player in activating autophagy (Qi et al., 2019; Sheng et al., 2021). This rare property of bufalin in inducing multiple ways of cell death, will endow it therapeutic implication in certain resistant cancer cells once they develop resistance to apoptosis, necroptosis or autophagy.

### Bufalin is effective as a chemo-sensitizer

**FIGURE 4:**
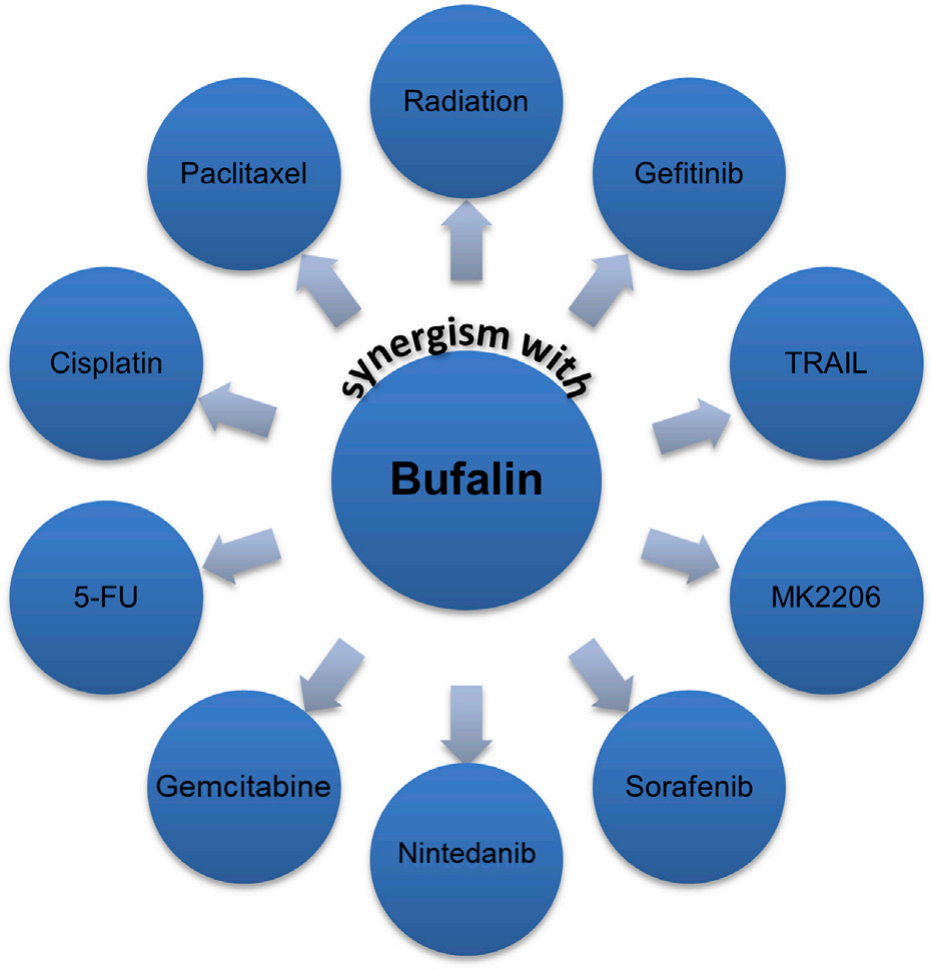
Bufalin has shown potential in working as a chemo-sensitizer.

Due to its natural toxic effect, many studies sought to use a low dose of bufalin to combine with other anticancer agents. Many promising results have been revealed that bufalin could enhance the sensitivity of a series of anticancer agents, including both conventional and targeted therapies, such as cisplatin, 5-FU, paclitaxel, adriamycin, gemcitabine, TRAIL, gefitinib, sorafenib, nintedanib, MK-2206 as shown in Figure 4 (Yan et al., 2014; Song et al., 2015; Sun et al., 2017; Wang et al., 2018a; Huang et al., 2018). However, the authors believe that bufalin’s role as a chemo-sensitizer has not been fully exploited. Thus, more efforts are encouraged to try combinational regimens containing bufalin.

### Bufalin is effective in inhibiting CSCs

CSCs are a subset of cancer cells with self-renewal ability (Shenouda et al., 2020; Carvalho et al., 2021). They play a critical role in cancer initiation and progression and are naturally resistant to anticancer agents, which cause cancer recurrence and MDR (Aravindan et al., 2019; Fong et al., 2021). Bufalin has been shown to effectively suppress CSCs from GBM, TNBC, pancreatic cancer, colorectal cancer, and osteosarcoma (Chang et al., 2015; Wang et al., 2016; Sun et al., 2017; Zhang et al., 2017; Chen et al., 2020a). Again, its complete therapeutic application remains to be explored.

### Bufalin works differently in inducing cell cycle arrest in different types of cancers

Cancer cells are avidly dividing, growing and proliferating, *etc.* Cell division is a dynamic and tightly regulated process by many essential proteins which can serve as a target for treatment (Liu et al., 2022). While bufalin effectively induces cell cycle arrest, it shows different patterns in different cell types. Bufalin could induce cell cycle arrest at the S phase in pancreatic cancer BxPC3-luc2 cells (Liu et al., 2016), lung cancer A549 cells (Zhang and Fu, 2017) at G2/M phase in GBM U251 and U87MG cells (Zhang et al., 2017), TNBC MDA-MB-231 and HCC-1937 cells (Chen et al., 2020b), pancreatic cancer PANC-1, and CFPAC-1 cells (Li et al., 2014), cervical cancer Siha cells (Liu et al., 2016). These results suggested that bufalin may interfere with cancer cell division, which can be further utilized with certain anticancer therapies.

### Current challenges

#### Toxic effects

The major challenge in using bufalin is the toxic effect, as 1) it targets Na^+^/K^+^-ATPase to exert its toxic effects towards toad’s enemy (Xie et al., 2001); 2) it can cause neuron toxicity via inhibiting voltage-gated potassium channels (Hao et al., 2011); 3) its median lethal dose (LD_50_) in nude mice is only 2.2 mg/kg (Tu et al., 2000), which is pretty close to the doses of achieving therapeutic effects of tumor inhibition (typically ∼1 mg/kg). The accumulated bufalin in blood in organs may cause severe adverse or toxic effects on normal tissues/organs. To use it more rationally, an in-depth animal model and human pharmacokinetic study are required to determine therapeutic windows and safe doses for certain cancerous patients.

#### Cytoprotective effects

Besides growing evidence supporting bufalin’s therapeutic effects in cancers, controversial studies also showed bufalin may promote cancers.

Chen et al. (2017) showed that in MB-231 breast cancer cells, bufalin (1 μM) could stimulate the inflammatory reaction induced by TPO, a protein kinase C (PKC) activator, leading to significantly higher levels of cyclooxygenase-2 (COX-2) and IL8, two inflammatory markers, and thereby a higher level of prostaglandin E_2_ (PGE_2_), which work together to stimulate MB-231 cell proliferation and invasion (Chen et al., 2017). Finally, bufalin (10 μL of 1 μM solution) could enhance tumor growth, accompanied by enhanced levels of COX-1 and IL8 (Chen et al., 2017). This study indicated that bufalin may stimulate an inflammatory response, promoting tumor growth, possibly through the upregulation of COX-2 and IL8. Interestingly, in this study, bufalin also could increase the expression of MMP3, an essential protein in regulating cell migration, which is in contrast to another study that showed bufalin reduced the levels of MMP2/9 in liver cancer HCCLM3 and HepG2 cells (Qiu et al., 2013). While very limited data show that bufalin could promote cancer growth, it should be cautious to monitor certain inflammatory factors when applied in humans.

#### Limited structure-activity relationship (SAR) information

**FIGURE 5:**
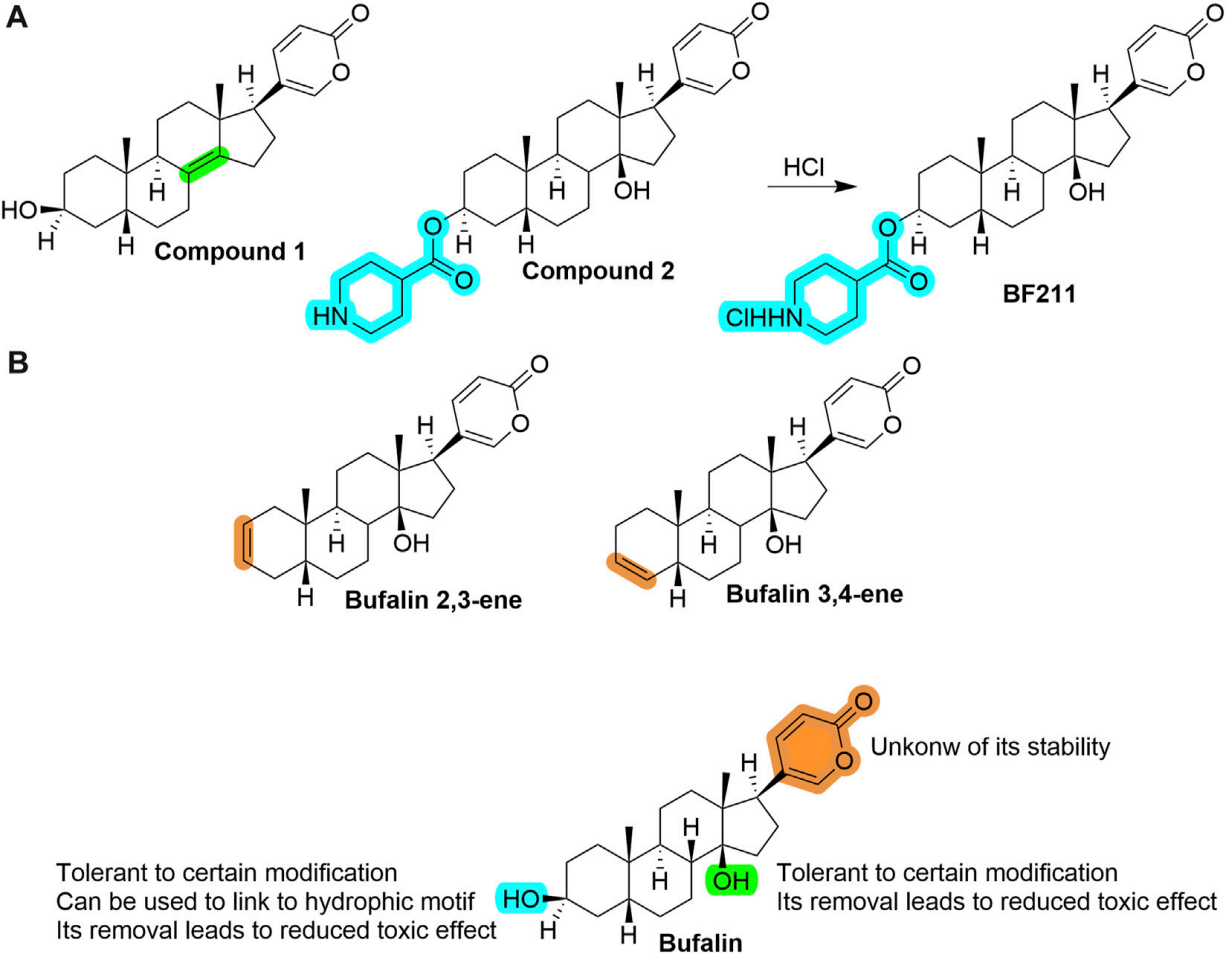
**(A)** Structures of several prominent bufalin derivatives. Significant structural properties are highlighted. **(B)** SAR information of bufalin by far (as of February 2023), and some remaining open questions to be answered.

By far, there is limited medicinal chemistry study based on bufalin, and no derivative shows a better cytotoxicity than bufalin. In addition to 3-phosphate-bufalin (Figure 2) (Song et al., 2015), there are several promising derivatives built on bufalin, including compound 1 (Yuan et al., 2014), compound 2 (Ma et al., 2013), compound BF211 (Lei et al., 2016; Sun et al., 2016), bufalin 2,3-ene and bufalin 3,4-ene (Sampath et al., 2022), *etc.*, As shown in Figure 5A. It is noticeable that most of them were modified on two hydroxyl groups. Unfortunately, those compounds all demonstrated much lower cytotoxicity than bufalin, while some had reduced effects toward Na^+^, K^+^-ATPase, suggesting a safer profile.

We extracted critical information in Figure 5B, which may help guide further structural modifications that aim to decrease toxicity yet maintain its cytotoxicity toward cancer cells (Shao et al., 2021).

### Future perspectives

#### In-depth pharmacological/mechanistic study

As discussed in Sections 2 and 3.1, bufalin appears more like a multi-targeting compound. It remains unknown 1) which target plays a leading or decisive role in killing cancer cells and what targets are simply down-stream pathways; 2) whether bufalin’s cytotoxicity is cancer type-dependent; 3) how the network of all targets works together to suppress cancer; *etc.* Thus, more studies of pharmacology and mechanism are needed to answer these questions.

#### Combination remedies

It has been confirmed that bufalin may achieve strong synergistic effects when combined with conventional and targeted therapies; however, more tryouts are worth evaluating, including its combination with immunotherapies. Bufalin has the potential to stimulate the immune response in NK cells and in inducing inflammatory cytokines (Shih et al., 2018; Fu et al., 2021), thus, it is rational to assume that it may have a synergistic effect with certain immunotherapies such as PD-1 or PD-L1 antibodies.

#### More *in vivo* models validation of bufalin

Translating *in vitro* into *in vivo* effects is challenging due to a different physiological environment. Thus, single-use or combinational remedies must be tried in animal models to reveal efficacies and potential toxic effects before evaluating cancerous patients in clinical trials.

#### A comprehensive and systematic SAR study of bufalin derivatives

To design new derivatives or analogs rationally, a complete picture of SAR is necessary, usually obtained from a comprehensive and systematic study that shows the relationship of each functional group with its activity. It has been shown that the two hydroxyl groups are either tolerant of being protected by certain groups through ester bond or be eliminated to generate a double bond product, which shows a lower inhibitory effect to Na+/K + -ATPase, the central origin of toxic effects (Ma et al., 2013; Yuan et al., 2014; Lei et al., 2016). Apparently, more information is needed to check if any other modifications are favorable, such as (1) ether bond linked with varied functional groups, (2) replacement with an imine bond linked with other groups, *etc.* As for the lactone ring, little information is known; open questions include whether it is stable under stocking conditions or whether it can undergo hydrolysis, which may cause the loss of activity of shorter half time. Thus, a more systematic study is needed for the rational drug design to improve its drug-likeness further and obtain a more suitable candidate, i.e., safe and effective, for evaluation in cancer patients.

## Conclusion

Bufalin is a highly cytotoxic agent that has been shown to inhibit a broad cancer type, including CSCs. Bufalin primarily targets and inhibits SRC-3, p-Akt, and the associated proteins in the corresponding pathways, leading to mitochondria-mediated apoptosis and necroptosis. While under some circumstances, these effects are cancer-type-dependent. Bufalin’s anticancer spectrum is not revealed entirely. Further in-depth study of pharmacology, medicinal chemistry, pharmacokinetics, and pharmacodynamics, *etc.*, are necessary to push bufalin as a drug candidate in clinical trials and eventually in patients.

## Figures and Tables

**Table:** 

**ABC**	ATP-binding cassette
**CSCs**	Cancer stem cells
**MDR**	Multi-drug resistance
**TCM**	Traditional Chinese medicine
**GBM**	Glioblastoma
**BBB**	Blood-brain barrier
**RIPK1/3**	Receptor-interacting serine/threonine-protein kinase 1/3
**MLKL**	Mixed lineage kinase domain-like protein
**TNF-α**	Tumor necrosis factor-α
**TNFR1**	TNF receptor 1
**IAP**	Inhibitor of Apoptosis Proteins
**ATP1A1**	Sodium pump alpha1 subunit
**H&E**	Hematoxylin and eosin
**IHC**	Immunohistochemistry
**PCNA**	Proliferating-cell nuclear antigen
**DSB**	Double-strand break
**XPO1**	Exportin 1
**SPARC**	Secreted protein acidic and rich in cysteine
**ER**	Endoplasmic reticulum
**ROS**	Reactive oxygen species
**ATF6**	Activating transcription factor 6
**PERK**	PKR-like ER kinase
**eIF2α**	Eukaryotic translation initiation factor 2α
**IRE1**	Inositol requiring enzyme 1
**CHOP**	C/EBP homologous protein
**AMPK**	AMP-activated protein kinase
**mTOR**	Mammalian target of rapamycin
**MMP**	Mitochondrial membrane potential
**HR**	Homologous recombination
**TNBC**	Triple-negative breast cancer
**HER2**	Human epidermal growth factor receptor 2
**PARP1**	(ADP-ribose) polymerase-1
**RIP1/3**	Receptor-interacting protein 1/3
**FOXO3a**	Factor forkhead box class O 3a
**DNMT1/3a**	DNA methyltransferases 1 and 3a
**SRC-3**	Steroid receptor coactivator 3
**EGFR**	Epidermal growth factor receptor
**TKIs**	Tyrosine kinase inhibitors
**TEM**	Transmission electron microscope
**TRAIL**	Tumor necrosis factor-related apoptosis-inducing ligand
**DR4**	Death receptor 4
**Cbla/b**	Casitas B-lineage lymphoma-a/b
**HCC**	Hepatocellular carcinoma
**MICA**	Major histocompatibility complex class I-related chain A
**MMP2/9**	Metalloproteinase 2/9
**GSK**	Glycogen synthase kinase
**P-gp**	P-glycoprotein
**MRP1**	Multi-drug resistance protein 1
**hTERT**	Human telomerase reverse transcriptase
**Hsp27**	Heat shock protein 27
**ASK1**	Apoptosis signal-regulating kinase 1
**ESA**	Epithelial specific antigen
**uPA**	Urokinase plasminogen activator
**FAK**	P-focal adhesion kinase
**TIMP1**	Tissue inhibitor matrix metalloproteinase 1
**HGF**	Hepatocyte growth factor
**CRC**	Colorectal cancer
**PDT**	Photodynamic therapy
**HIF-1α**	Hypoxia inducible factor 1α
**SP**	Side-population
**ADT**	Androgen deprivation therapy
**Top1**	Topoisomerase I
**CRPC**	Castration-resistant prostate cancer
**PKC**	Protein kinase C
**COX-2**	Cyclooxygenase-2
**PGE2**	Prostaglandin E2

## References

[B1] Aravindan N. Jain D. Somasundaram D. B. Herman T. S. Aravindan S. (2019). Cancer stem cells in neuroblastoma therapy resistance. Cancer Drug Resist 2, 948–967. 10.20517/cdr.2019.72 31867574 PMC6924637

[B2] Ashrafi A. Akter Z. Modareszadeh P. Modareszadeh P. Berisha E. Alemi P. S. (2022). Current landscape of therapeutic resistance in lung cancer and promising strategies to overcome resistance. Cancers 14, 4562. 10.3390/cancers14194562 36230484 PMC9558974

[B3] Beebe J. Josephraj S. Wang C. J. Danielson J. Cui Q. Huang C. (2022). Therapeutic activity of the lansoprazole metabolite 5-hydroxy lansoprazole sulfide in triple-negative breast cancer by inhibiting the enoyl reductase of fatty acid synthase. J. Med. Chem. 65, 13681–13691. 10.1021/acs.jmedchem.2c00642 36257066

[B4] Bengtsson A. Andersson R. Ansari D. (2020). The actual 5-year survivors of pancreatic ductal adenocarcinoma based on real-world data. Sci. Rep-Uk 10, 16425. 10.1038/s41598-020-73525-y PMC7532215 33009477

[B5] Bielack S. S. Blattmann C. Borkhardt A. Csóka M. Hassenpflug W. Kabíčková E. (2022), Osteosarcoma and causes of death: A report of 1520 deceased patients from the cooperative osteosarcoma study group (COSS). Eur. J. Cancer 176, 50–57. 10.1016/j.ejca.2022.09.007 36191386

[B6] Carvalho L. S. Goncalves N. Fonseca N. A. Moreira J. N. (2021). Cancer stem cells and nucleolin as drivers of carcinogenesis. Pharmaceuticals-Base 14, 60. 10.3390/ph14010060 PMC7828541 33451077

[B7] Chang Y. Zhao Y. Gu W. Cao Y. Wang S. Pang J. (2015). Bufalin inhibits the differentiation and proliferation of cancer stem cells derived from primary osteosarcoma cells through mir-148a. Cell. Physiol. Biochem. 36, 1186–1196. 10.1159/000430289 26111756

[B8] Chen B. Chen C. Zhang Y. Xu J. (2021a). Recent incidence trend of elderly patients with glioblastoma in the United States, 2000-2017. Bmc Cancer 21, 54. 10.1186/s12885-020-07778-1 33430813 PMC7802341

[B9] Chen F. Zhu L. Hu J. Jiang S. Liu H. Zheng J. (2020a). Bufalin attenuates triple-negative breast cancer cell stemness by inhibiting the expression of SOX2/OCT4. Oncol. Lett. 20, 171. 10.3892/ol.2020.12028 32934738 PMC7471667

[B10] Chen H. T. Sun D. Peng Y. C. Kao P. H. Wu Y. L. (2017). Novel augmentation by bufalin of protein kinase C-induced cyclooxygenase-2 and IL-8 production in human breast cancer cells. Innate Immun-London 23, 54–66. 10.1177/1753425916676347 27821648

[B11] Chen J. G. Zhu J. Zhang Y. H. Chen Y. S. Ding L. L. Chen H. Z. (2021b). Liver cancer survival: A real world observation of 45 Years with 32,556 cases. J. Hepatocell. Carcino 8, 1023–1034. 10.2147/JHC.S321346 PMC8418373 34513745

[B12] Chen J. Wang H. Jia L. He J. Li Y. Liu H. (2021c). Bufalin targets the SRC-3/MIF pathway in chemoresistant cells to regulate M2 macrophage polarization in colorectal cancer. Cancer Lett. 513, 63–74. 10.1016/j.canlet.2021.05.008 34000344

[B13] Chen L. Yang F. Chen S. Tai J. (2022). Mechanisms on chemotherapy resistance of colorectal cancer stem cells and research progress of reverse transformation: A mini-review. Front. Med-Lausanne 9, 995882. 10.3389/fmed.2022.995882 36172536 PMC9510709

[B14] Chen Y. Guo Q. Zhang B. Kang M. Xie Q. Wu Y. (2012). Bufalin enhances the antitumor effect of gemcitabine in pancreatic cancer. Oncol. Lett. 4, 792–798. 10.3892/ol.2012.783 23205102 PMC3506674

[B15] Chen Y. L. Dai Y. H. Wang A. D. Zhou Z. Y. Lei M. Liu J. (2020b). Two new indole alkaloids from toad venom of *Bufo bufo* gargarizans. Molecules 25, 4511. 10.3390/molecules25194511 33019706 PMC7582642

[B16] Chien A. J. Tripathy D. Albain K. S. Symmans W. F. Rugo H. S. Melisko M. E. (2020). MK-2206 and standard neoadjuvant chemotherapy improves response in patients with human epidermal growth factor receptor 2-positive and/or hormone receptor-negative breast cancers in the I-spy 2 trial. J. Clin. Oncol. 38, 1059–1069. 10.1200/JCO.19.01027 32031889 PMC7106976

[B17] Choi J. Y. Yeob K. E. Hong S. H. Kim S. Y. Jeong E. H. Shin D. W. (2021). Disparities in the diagnosis, treatment, and survival rate of cervical cancer among women with and without disabilities. Cancer control 28, 10732748211055268. 10.1177/10732748211055268 35042390 PMC8771753

[B18] Cui Q. Liang X. L. Wang J. Q. Zhang J. Y. Chen Z. S. (2022). Therapeutic implication of carbon monoxide in drug resistant cancers. Biochem. Pharmacol. 201, 115061. 10.1016/j.bcp.2022.115061 35489394

[B19] Cui Q. Wang J. Q. Assaraf Y. G. Ren L. Gupta P. Wei L. (2018). Modulating ROS to overcome multidrug resistance in cancer. Drug resist. Update 41, 1–25. 10.1016/j.drup.2018.11.001 30471641

[B20] Dai X. Y. Zhou B. F. Xie Y. Y. Lou J. Li K. Q. (2018a). Bufalin and 5-fluorouracil synergistically induce apoptosis in colorectal cancer cells. Oncol. Lett. 15, 8019–8026. 10.3892/ol.2018.8332 29849804 PMC5962859

[B21] Dai Y. H. Wang A. D. Chen Y. L. Xia M. Y. Shao X. Y. Liu D. C. (2018b). A new indole alkaloid from the traditional Chinese medicine Chansu. J. Asian Nat. Prod. Res. 20, 581–585. 10.1080/10286020.2017.1339697 28625094

[B22] Dong Q. Liu L. Yuan Y. Turdu G. Mirzaakhmedov S. Aisa H. A. (2022a). Two new polyamine alkaloids from the *Bufo viridis* toad venom. Nat. Prod. Res. 1-5, 3538–3542. 10.1080/14786419.2022.2086545 35675547

[B23] Dong X. D. Zhang M. Cai C. Y. Teng Q. X. Wang J. Q. Fu Y. G. (2022b). Overexpression of ABCB1 associated with the resistance to the KRAS-G12C specific inhibitor ARS-1620 in cancer cells. Front. Pharmacol. 13, 843829. 10.3389/fphar.2022.843829 35281897 PMC8905313

[B24] Feng X. Y. Zhao W. Yao Z. Wei N. Y. Shi A. H. Chen W. H. (2021). Downregulation of ATP1A1 expression by panax notoginseng (burk.) F.H. Chen saponins: A potential mechanism of antitumor effects in HepG2 cells and *in vivo* . Front. Pharmacol. 12, 720368. 10.3389/fphar.2021.720368 34690763 PMC8529207

[B25] Ferlay J. Colombet M. Soerjomataram I. Parkin D. M. Pineros M. Znaor A. (2021). Estimating the global cancer incidence and mortality in 2018: GLOBOCAN sources and methods. Int. J. Cancer. 144, 1941–1953. 10.1002/ijc.31937 30350310

[B26] Fong D. Christensen C. T. Chan M. M. (2021). Targeting cancer stem cells with repurposed drugs to improve current therapies. Recent Pat. Anti-Canc 16, 136–160. 10.2174/1574892816666210208232251 33563159

[B27] Fu J. Su X. Li Z. Deng L. Liu X. Feng X. (2021). HGF/c-MET pathway in cancer: From molecular characterization to clinical evidence. Oncogene 40, 4625–4651. 10.1038/s41388-021-01863-w 34145400

[B28] Fu R. Yu F. Wu W. Liu J. Li J. Guo F. (2021). Bufalin enhances the killing efficacy of NK cells against hepatocellular carcinoma by inhibiting MICA shedding. Int. Immunopharmacol. 101, 108195. 10.1016/j.intimp.2021.108195 34678691

[B29] Fulda S. (2009). Tumor resistance to apoptosis. Int. J. Cancer 124, 511–515. 10.1002/ijc.24064 19003982

[B30] Gao Y. Li H. X. Xu L. T. Wang P. Xu L. Y. Cohen L. (2012). Bufalin enhances the anti-proliferative effect of sorafenib on human hepatocellular carcinoma cells through downregulation of ERK. Mol. Biol. Rep. 39, 1683–1689. 10.1007/s11033-011-0908-x 21617941

[B31] Giaquinto A. N. Sung H. Miller K. D. Kramer J. L. Newman L. A. Minihan A. (2022). Breast cancer statistics, 2022. Ca-Cancer J. Clin. 72, 524–541. 10.3322/caac.21754 36190501

[B32] Global (2019). Global, regional, and national burden of brain and other CNS cancer, 1990-2016: A systematic analysis for the global burden of disease study 2016. Lancet Neurol. 18, 376–393. 10.1016/S1474-4422(18)30468-X 30797715 PMC6416167

[B33] Gu R. Zhang Q. (2021). Effects of low-dose bufalin combined with hydroxycamptothecin on human castration-resistant prostate cancer xenografts in nude mice. Exp. Ther. Med. 22, 1015. 10.3892/etm.2021.10447 34373701 PMC8343571

[B34] Gu W. Liu L. Fang F. F. Huang F. Cheng B. B. Li B. (2014). Reversal effect of bufalin on multidrug resistance in human hepatocellular carcinoma BEL-7402/5-FU cells. Oncol. Rep. 31, 216–222. 10.3892/or.2013.2817 24173654

[B35] Hao S. Bao Y. M. An L. J. Cheng W. Zhao R. G. Bi J. (2011). Effects of Resibufogenin and Cinobufagin on voltage-gated potassium channels in primary cultures of rat hippocampal neurons. Toxicol. Vitro 25, 1644–1653. 10.1016/j.tiv.2011.07.001 21798339

[B36] Hayes J. D. Dinkova-Kostova A. T. Tew K. D. (2020). Oxidative stress in cancer. Cancer Cell. 38, 167–197. 10.1016/j.ccell.2020.06.001 32649885 PMC7439808

[B37] Hong S. H. Choi Y. H. (2012). Bufalin induces apoptosis through activation of both the intrinsic and extrinsic pathways in human bladder cancer cells. Oncol. Rep. 27, 114–120. 10.3892/or.2011.1451 21901250

[B38] Hu T. Li Z. Gao C. Y. Cho C. H. (2016). Mechanisms of drug resistance in colon cancer and its therapeutic strategies. World J. gastroentero. 22, 6876–6889. 10.3748/wjg.v22.i30.6876 PMC4974586 27570424

[B39] Huang A. C. Yang M. D. Hsiao Y. T. Lin T. S. Ma Y. S. Peng S. F. (2016). Bufalin inhibits gefitinib resistant NCI-H460 human lung cancer cell migration and invasion *in vitro* . J. Ethnopharmacol. 194, 1043–1050. 10.1016/j.jep.2016.11.004 27833027

[B40] Huang H. Lin X. J. Lin Y. Yao R. X. He M. Q. (2018). Bufalin enhances the cytotoxity of human multiple myeloma cells H929 to AKT inhibitor MK2206: The role of protein AKT phosphorylation. Indian J. Hematol. Blo 34, 268–272. 10.1007/s12288-017-0883-z PMC5884994 29622868

[B41] Huang X. Li E. Shen H. Wang X. Tang T. Zhang X. (2020). Targeting the HGF/MET Axis in cancer therapy: Challenges in resistance and opportunities for improvement. Front. Cell. Dev. Biol. 8, 152. 10.3389/fcell.2020.00152 32435640 PMC7218174

[B42] Jiang M. Jia K. Wang L. Li W. Chen B. Liu Y. (2020). Alterations of DNA damage repair in cancer: From mechanisms to applications. Ann. Transl. Med. 8, 1685. 10.21037/atm-20-2920 33490197 PMC7812211

[B43] Kang K. H. Han M. H. Jeong J. W. Park C. Lee S. H. Lee H. W. (2017). Bufalin sensitizes human bladder carcinoma cells to TRAIL-mediated apoptosis. Oncol. Lett. 14, 853–859. 10.3892/ol.2017.6223 28693242 PMC5494769

[B44] Kang X. H. Xu Z. Y. Gong Y. B. Wang L. F. Wang Z. Q. Xu L. (2013). Bufalin reverses HGF-induced resistance to EGFR-TKIs in EGFR mutant lung cancer cells via blockage of Met/PI3k/akt pathway and induction of apoptosis. Evid-Based Compl Alt. 2013, 243859. 10.1155/2013/243859 PMC3603503 23533466

[B45] Kang X. Lu P. Cui Y. Wang Y. Zhang Q. Gong Y. (2015). Bufalin reverses hepatocyte growth factor-induced resistance to afatinib in H1975 lung cancer cells. Zhonghua Zhong Liu Za Zhi 37, 490–496. 26463323

[B46] Kathawala R. J. Gupta P. Ashby C. J. Chen Z. S. (2015). The modulation of ABC transporter-mediated multidrug resistance in cancer: A review of the past decade. Drug resist. Update 18, 1–17. 10.1016/j.drup.2014.11.002 25554624

[B47] Kohale I. N. Yu J. Zhuang Y. Fan X. Reddy R. J. Sinnwell J. (2022). Identification of src family kinases as potential therapeutic targets for chemotherapy-resistant triple negative breast cancer. Cancers 14, 4220. 10.3390/cancers14174220 36077757 PMC9454481

[B48] Kuo J. Y. Liao C. L. Ma Y. S. Kuo C. L. Chen J. C. Huang Y. P. (2022). Combination treatment of sorafenib and bufalin induces apoptosis in NCI-H292 human lung cancer cells *in vitro* . Vivo 36, 582–595. 10.21873/invivo.12741 PMC8931928 35241510

[B49] Lan Y. L. Wang X. Lou J. C. Xing J. S. Yu Z. L. Wang H. (2018). Bufalin inhibits glioblastoma growth by promoting proteasomal degradation of the Na+/K+-ATPase α1 subunit. Biomed. Pharmacother. 103, 204–215. 10.1016/j.biopha.2018.04.030 29653366

[B50] Lan Y. L. Zou Y. J. Lou J. C. Xing J. S. Wang X. Zou S. (2019). The sodium pump α1 subunit regulates bufalin sensitivity of human glioblastoma cells through the p53 signaling pathway. Cell. Biol. Toxicol. 35, 521–539. 10.1007/s10565-019-09462-y 30739221

[B51] Laursen M. Gregersen J. L. Yatime L. Nissen P. Fedosova N. U. (2015). Structures and characterization of digoxin- and bufalin-bound Na+,K+-ATPase compared with the ouabain-bound complex. P. Natl. Acad. Sci. U. S. A. 112, 1755–1760. 10.1073/pnas.1422997112 PMC4330780 25624492

[B52] Lei M. Xiao Z. Ma B. Chen Y. Liu M. Liu J. (2016), Synthesis and biological evaluation of bufalin-3-yl nitrogen-containing-carbamate derivatives as anticancer agents. Steroids 108, 56–60. 10.1016/j.steroids.2016.01.011 26827628

[B53] Li D. Qu X. Hou K. Zhang Y. Dong Q. Teng Y. (2009). PI3K/Akt is involved in bufalin-induced apoptosis in gastric cancer cells. Anti-Cancer Drug 20, 59–64. 10.1097/CAD.0b013e3283160fd6 19343001

[B54] Li F. J. Hu J. H. Ren X. Zhou C. M. Liu Q. Zhang Y. Q. (2021). Toad venom: A comprehensive review of chemical constituents, anticancer activities, and mechanisms. Arch. Pharm. 354, e2100060. 10.1002/ardp.202100060 33887066

[B55] Li L. Y. Guan Y. D. Chen X. S. Yang J. M. Cheng Y. (2020). DNA repair pathways in cancer therapy and resistance. Front. Pharmacol. 11, 629266. 10.3389/fphar.2020.629266 33628188 PMC7898236

[B56] Li M. Yu X. Guo H. Sun L. Wang A. Liu Q. (2014). Bufalin exerts antitumor effects by inducing cell cycle arrest and triggering apoptosis in pancreatic cancer cells. Tumour Biol. 35, 2461–2471. 10.1007/s13277-013-1326-6 24218335

[B57] Li W. Zhang H. Assaraf Y. G. Zhao K. Xu X. Xie J. (2016). Overcoming ABC transporter-mediated multidrug resistance: Molecular mechanisms and novel therapeutic drug strategies. Drug resist. Update 27, 14–29. 10.1016/j.drup.2016.05.001 27449595

[B58] Li Y. J. Lei Y. H. Yao N. Wang C. R. Hu N. Ye W. C. (2017). Autophagy and multidrug resistance in cancer. Chin. J. Cancer 36, 52. 10.1186/s40880-017-0219-2 28646911 PMC5482965

[B59] Li Y. Tian X. Liu X. Gong P. (2018). Bufalin inhibits human breast cancer tumorigenesis by inducing cell death through the ROS-mediated RIP1/RIP3/PARP-1 pathways. Carcinogenesis 39, 700–707. 10.1093/carcin/bgy039 29546393

[B60] Li Y. Zhang H. Merkher Y. Chen L. Liu N. Leonov S. (2022). Recent advances in therapeutic strategies for triple-negative breast cancer. J. Hematol. Oncol. 15, 121. 10.1186/s13045-022-01341-0 36038913 PMC9422136

[B61] LingHu H. R. Luo H. Gang L. (2020). Bufalin induces glioma cell death by apoptosis or necroptosis. Oncotargets Ther. 13, 4767–4778. 10.2147/OTT.S242567 PMC7274536 32581545

[B62] Liu F. Tong D. Li H. Liu M. Li J. Wang Z. (2016). Bufalin enhances antitumor effect of paclitaxel on cervical tumorigenesis via inhibiting the integrin α2/β5/FAK signaling pathway. Oncotarget 7, 8896–8907. 10.18632/oncotarget.6840 26758421 PMC4891012

[B63] Liu J. Peng Y. Wei W. (2022). Cell cycle on the crossroad of tumorigenesis and cancer therapy. Trends Cell. Biol. 32, 30–44. 10.1016/j.tcb.2021.07.001 34304958 PMC8688170

[B64] Liu T. Wu C. Weng G. Zhao Z. He X. Fu C. (2017). Bufalin inhibits cellular proliferation and cancer stem cell-like phenotypes via upregulation of MiR-203 in glioma. Cell. Physiol. Biochem. 44, 671–681. 10.1159/000485279 29169175

[B65] Liu X. D. Song C. Y. Kong C. C. Tian X. (2021). Bufalin induces programmed necroptosis in triple-negative breast cancer drug-resistant cell lines through RIP1/ROS-mediated pathway. Chin. J. Integr. Med. 28, 900–908. 10.1007/s11655-021-3458-7 34826043

[B66] Liu X. Xiao X. Y. Shou Q. Y. Yan J. F. Chen L. Fu H. Y. (2016). Bufalin inhibits pancreatic cancer by inducing cell cycle arrest via the c-Myc/NF-κB pathway. J. Ethnopharmacol. 193, 538–545. 10.1016/j.jep.2016.09.047 27686271

[B67] Ma B. Xiao Z. Chen Y. Lei M. Meng Y. Guo D. (2013), Synthesis and structure–activity relationships study of cytotoxic bufalin 3-nitrogen-containing-ester derivatives. Steroids 78, 508–512. 10.1016/j.steroids.2013.02.007 23485688

[B68] Meng Q. Yau L. F. Lu J. G. Wu Z. Z. Zhang B. X. Wang J. R. (2016). Chemical profiling and cytotoxicity assay of bufadienolides in toad venom and toad skin. J. Ethnopharmacol. 187, 74–82. 10.1016/j.jep.2016.03.062 27063985

[B69] Misaghi A. Goldin A. Awad M. Kulidjian A. A. (2018). Osteosarcoma: A comprehensive review. Sicot-J 4, 12. 10.1051/sicotj/2017028 29629690 PMC5890448

[B70] Miyoshi N. Haraguchi N. Mizushima T. Ishii H. Yamamoto H. Mori M. (2021). Targeting cancer stem cells in refractory cancer. Regen. Ther. 17, 13–19. 10.1016/j.reth.2021.01.002 33598510 PMC7868918

[B71] Morgan M. J. Kim Y. S. (2022). Roles of RIPK3 in necroptosis, cell signaling, and disease. Exp. Mol. Med. 54, 1695–1704. 10.1038/s12276-022-00868-z 36224345 PMC9636380

[B72] Mulcahy L. J. Thorburn A. (2020). Autophagy in cancer: Moving from understanding mechanism to improving therapy responses in patients. Cell. Death Differ. 27, 843–857. 10.1038/s41418-019-0474-7 31836831 PMC7206017

[B73] Nakazawa M. Paller C. Kyprianou N. (2017). Mechanisms of therapeutic resistance in prostate cancer. Curr. Oncol. Rep. 19, 13. 10.1007/s11912-017-0568-7 28229393 PMC5812366

[B74] Narayanan S. Cai C. Y. Assaraf Y. G. Guo H. Q. Cui Q. Wei L. (2020). Targeting the ubiquitin-proteasome pathway to overcome anti-cancer drug resistance. Drug resist. Update 48, 100663. 10.1016/j.drup.2019.100663 31785545

[B75] Narayanan S. Gujarati N. A. Wang J. Q. Wu Z. X. Koya J. Cui Q. (2021). The novel benzamide derivative, VKNG-2, restores the efficacy of chemotherapeutic drugs in colon cancer cell lines by inhibiting the ABCG2 transporter. Int. J. Mol. Sci. 22, 2463. 10.3390/ijms22052463 33671108 PMC7957563

[B76] Neophytou C. M. Trougakos I. P. Erin N. Papageorgis P. (2021). Apoptosis deregulation and the development of cancer multi-drug resistance. Cancers 13, 4363. 10.3390/cancers13174363 34503172 PMC8430856

[B77] Oki Y. Fanale M. Romaguera J. Fayad L. Fowler N. Copeland A. (2015). Phase II study of an AKT inhibitor MK2206 in patients with relapsed or refractory lymphoma. Brit. J. Haematol. 171, 463–470. 10.1111/bjh.13603 26213141 PMC5278973

[B78] Okon I. S. Zou M. H. (2015). Mitochondrial ROS and cancer drug resistance: Implications for therapy. Pharmacol. Res. 100, 170–174. 10.1016/j.phrs.2015.06.013 26276086 PMC4893310

[B79] Pagliarini R. Shao W. Sellers W. R. (2015). Oncogene addiction: Pathways of therapeutic response, resistance, and road maps toward a cure. Embo Rep. 16, 280–296. 10.15252/embr.201439949 25680965 PMC4364868

[B80] Peery R. Cui Q. Kyei-Baffour K. Josephraj S. Huang C. Dong Z. (2022). A novel survivin dimerization inhibitor without a labile hydrazone linker induces spontaneous apoptosis and synergizes with docetaxel in prostate cancer cells. Bioorgan. Med. Chem. 65, 116761. 10.1016/j.bmc.2022.116761 PMC9148172 35504208

[B81] Petrylak D. P. (2013). Current state of castration-resistant prostate cancer. Am. J. Manag. Care 19, s358–s365. 24494690

[B82] Qi H. Y. Qu X. J. Liu J. Hou K. Z. Fan Y. B. Che X. F. (2019). Bufalin induces protective autophagy by Cbl-b regulating mTOR and ERK signaling pathways in gastric cancer cells. Cell. Biol. Int. 43, 33–43. 10.1002/cbin.11076 30468278

[B83] Qiu D. Z. Zhang Z. J. Wu W. Z. Yang Y. K. (2013). Bufalin, a component in Chansu, inhibits proliferation and invasion of hepatocellular carcinoma cells. Bmc Complem Altern. M. 13, 185. 10.1186/1472-6882-13-185 PMC3723921 23870199

[B84] Qu T. Gao H. M. Chen L. M. Wang Z. M. Zhang Q. W. Cheng Y. Y. (2012). Content of indole alkaloids and bufadienolides contained in toad medicines. Zhongguo Zhong Yao Za Zhi 37, 3086–3091. 23311159

[B85] Rawla P. (2019). Epidemiology of prostate cancer. World J. Oncol. 10, 63–89. 10.14740/wjon1191 31068988 PMC6497009

[B86] Rawla P. Sunkara T. Gaduputi V. (2019). Epidemiology of pancreatic cancer: Global trends, etiology and risk factors. World J. Oncol. 10, 10–27. 10.14740/wjon1166 30834048 PMC6396775

[B87] Reuvers T. Kanaar R. Nonnekens J. (2020). DNA damage-inducing anticancer therapies: From global to precision damage. Cancers 12, 2098. 10.3390/cancers12082098 32731592 PMC7463878

[B88] Robey R. W. Pluchino K. M. Hall M. D. Fojo A. T. Bates S. E. Gottesman M. M. (2018). Revisiting the role of ABC transporters in multidrug-resistant cancer. Nat. Rev. Cancer 18, 452–464. 10.1038/s41568-018-0005-8 29643473 PMC6622180

[B89] Rodriguez C. Ibanez R. Ng M. Spadafora C. Durant-Archibold A. A. Gutierrez M. (2020). 19-Hydroxy-bufalin, a major bufadienolide isolated from the parotoid gland secretions of the Panamanian endemic toad Rhinella centralis (Bufonidae), inhibits the growth of Trypanosoma cruzi. Toxicon 177, 89–92. 10.1016/j.toxicon.2020.02.009 32061723

[B90] Rong X. Ni W. Liu Y. Wen J. Qian C. Sun L. (2014). Bufalin, a bioactive component of the Chinese medicine chansu, inhibits inflammation and invasion of human rheumatoid arthritis fibroblast-like synoviocytes. Inflammation 37, 1050–1058. 10.1007/s10753-014-9828-y 24515724

[B91] Rosell R. (2013). Mediating resistance in oncogene-driven cancers. New Engl. J. Med. 368, 1551–1552. 10.1056/NEJMcibr1214549 23594009

[B92] Rumgay H. Arnold M. Ferlay J. Lesi O. Cabasag C. J. Vignat J. (2022). Global burden of primary liver cancer in 2020 and predictions to 2040. J. Hepatol. 77, 1598–1606. 10.1016/j.jhep.2022.08.021 36208844 PMC9670241

[B93] Sajid A. Rahman H. Ambudkar S. V. (2023). Advances in the structure, mechanism and targeting of chemoresistance-linked ABC transporters. Nat. Rev. Cancer. 10.1038/s41568-023-00612-3 37714963

[B94] Sampath V. Horesh N. Sasi B. Zannadeh H. Pogodin I. Singh S. V. (2022). Synthesis and biological evaluation of novel bufalin derivatives. Int. J. Mol. Sci. 23, 4007. 10.3390/ijms23074007 35409366 PMC8999407

[B95] Sarkaria J. N. Hu L. S. Parney I. F. Pafundi D. H. Brinkmann D. H. Laack N. N. (2018). Is the blood-brain barrier really disrupted in all glioblastomas? A critical assessment of existing clinical data. Neuro-Oncology 20, 184–191. 10.1093/neuonc/nox175 29016900 PMC5777482

[B96] Shao H. Li B. Li H. Gao L. Zhang C. Sheng H. (2021). Novel strategies for solubility and bioavailability enhancement of bufadienolides. Molecules 27, 51. 10.3390/molecules27010051 35011278 PMC8746454

[B97] Shen S. Zhang Y. Wang Z. Liu R. Gong X. (2014). Bufalin induces the interplay between apoptosis and autophagy in glioma cells through endoplasmic reticulum stress. Int. J. Biol. Sci. 10, 212–224. 10.7150/ijbs.8056 24550689 PMC3927133

[B98] Sheng X. Zhu P. Zhao Y. Zhang J. Li H. Zhao H. (2021). Effect of PI3K/AKT/mTOR signaling pathway on regulating and controlling the anti-invasion and metastasis of hepatoma cells by bufalin. Recent Pat. Anti-Canc 16, 54–65. 10.2174/1574892816666210201120324 33530915

[B99] Shenouda S. Kulkarni K. Abuetabh Y. Sergi C. (2020). Cancer stem cells and their management in cancer therapy. Recent Pat. Anti-Canc 15, 212–227. 10.2174/1574892815666200713145931 32660407

[B100] Shih Y. L. Chou J. S. Chen Y. L. Hsueh S. C. Chung H. Y. Lee M. H. (2018). Bufalin enhances immune responses in leukemic mice through enhancing phagocytosis of macrophage *in vivo* . Vivo 32, 1129–1136. 10.21873/invivo.11355 PMC6199617 30150435

[B101] Shin D. S. Zaretsky J. M. Escuin-Ordinas H. Garcia-Diaz A. Hu-Lieskovan S. Kalbasi A. (2017). Primary resistance to PD-1 blockade mediated by JAK1/2 mutations. Cancer Discov. 7, 188–201. 10.1158/2159-8290.CD-16-1223 27903500 PMC5296316

[B102] Siegel R. L. Miller K. D. Goding S. A. Fedewa S. A. Butterly L. F. Anderson J. C. (2020). Colorectal cancer statistics, 2020. Ca-Cancer J. Clin. 70, 145–164. 10.3322/caac.21601 32133645

[B103] Simonds E. F. Lu E. D. Badillo O. Karimi S. Liu E. V. Tamaki W. (2021). Deep immune profiling reveals targetable mechanisms of immune evasion in immune checkpoint inhibitor-refractory glioblastoma. J. Immunother. Cancer 9, e002181. 10.1136/jitc-2020-002181 34083417 PMC8183210

[B104] Song X. Zhang C. Zhao M. Chen H. Liu X. Chen J. (2015). Steroid receptor coactivator-3 (SRC-3/AIB1) as a novel therapeutic target in triple negative breast cancer and its inhibition with a phospho-bufalin prodrug. Plos One 10, e0140011. 10.1371/journal.pone.0140011 26431029 PMC4592245

[B105] Su C. (2022). Emerging insights to lung cancer drug resistance. Cancer Drug Resist 5, 534–540. 10.20517/cdr.2022.61 36176761 PMC9511798

[B106] Sun J. Xu K. Qiu Y. Gao H. Xu J. Tang Q. (2017). Bufalin reverses acquired drug resistance by inhibiting stemness in colorectal cancer cells. Oncol. Rep. 38, 1420–1430. 10.3892/or.2017.5826 28731184 PMC5549034

[B107] Sun P. Feng L. X. Zhang D. M. Liu M. Liu W. Mi T. (2016). Bufalin derivative BF211 inhibits proteasome activity in human lung cancer cells *in vitro* by inhibiting β1 subunit expression and disrupting proteasome assembly. Acta Pharmacol. Sin. 37, 908–918. 10.1038/aps.2016.30 27238210 PMC4933757

[B108] Sung H. Ferlay J. Siegel R. L. Laversanne M. Soerjomataram I. Jemal A. (2021). Global cancer statistics 2020: GLOBOCAN estimates of incidence and mortality worldwide for 36 cancers in 185 countries. Ca-Cancer J. Clin. 71, 209–249. 10.3322/caac.21660 33538338

[B109] Takai N. Ueda T. Nishida M. Nasu K. Narahara H. (2008). Bufalin induces growth inhibition, cell cycle arrest and apoptosis in human endometrial and ovarian cancer cells. Int. J. Mol. Med. 21, 637–643. 10.3892/ijmm.21.5.637 18425357

[B110] Thomas C. Tampe R. (2020). Structural and mechanistic principles of ABC transporters. Annu. Rev. Biochem. 89, 605–636. 10.1146/annurev-biochem-011520-105201 32569521

[B111] Tian X. Dai S. Sun J. Jiang S. Sui C. Meng F. (2015). Bufalin induces mitochondria-dependent apoptosis in pancreatic and oral cancer cells by downregulating hTERT expression via activation of the JNK/p38 pathway. Evid-Based Compl Alt. 2015, 546210. 10.1155/2015/546210 PMC4689913 26783410

[B112] Tryfonopoulos D. Walsh S. Collins D. M. Flanagan L. Quinn C. Corkery B. (2011). Src: A potential target for the treatment of triple-negative breast cancer. Ann. Oncol. 22, 2234–2240. 10.1093/annonc/mdq757 21357651

[B113] Tsai S. C. Lu C. C. Lee C. Y. Lin Y. C. Chung J. G. Kuo S. C. (2012). AKT serine/threonine protein kinase modulates bufalin-triggered intrinsic pathway of apoptosis in CAL 27 human oral cancer cells. Int. J. Oncol. 41, 1683–1692. 10.3892/ijo.2012.1605 22922805

[B114] Tu S. P. Zhong J. Tan J. H. Jiang X. H. Qiao M. M. Wu Y. X. (2000). Induction of apoptosis by arsenic trioxide and hydroxy camptothecin in gastriccancer cells *in vitro* . World J. gastroentero. 6, 532–539. 10.3748/wjg.v6.i4.532 PMC4723552 11819642

[B115] Tumbrink H. L. Heimsoeth A. Sos M. L. (2021). The next tier of EGFR resistance mutations in lung cancer. Oncogene 40, 1–11. 10.1038/s41388-020-01510-w 33060857

[B116] van Tellingen O. Yetkin-Arik B. de Gooijer M. C. Wesseling P. Wurdinger T. de Vries H. E. (2015). Overcoming the blood-brain tumor barrier for effective glioblastoma treatment. Drug resist. Update 19, 1–12. 10.1016/j.drup.2015.02.002 25791797

[B117] Wang H. Ning Z. Li Y. Zhu X. Meng Z. (2016). Bufalin suppresses cancer stem-like cells in gemcitabine-resistant pancreatic cancer cells via Hedgehog signaling. Mol. Med. Rep. 14, 1907–1914. 10.3892/mmr.2016.5471 27432228 PMC4991682

[B118] Wang H. Wang Q. Cai G. Duan Z. Nugent Z. Huang J. (2022). Nuclear TIGAR mediates an epigenetic and metabolic autoregulatory loop via NRF2 in cancer therapeutic resistance. Acta Pharm. Sin. B 12, 1871–1884. 10.1016/j.apsb.2021.10.015 35847493 PMC9279715

[B119] Wang H. Zhang C. Chi H. Meng Z. (2018b). Synergistic anti-hepatoma effect of bufalin combined with sorafenib via mediating the tumor vascular microenvironment by targeting mTOR/VEGF signaling. Int. J. Oncol. 52, 2051–2060. 10.3892/ijo.2018.4351 29620259

[B120] Wang H. Zhang C. Chi H. Meng Z. (2018a). Synergistic anticancer effects of bufalin and sorafenib by regulating apoptosis associated proteins. Mol. Med. Rep. 17, 8101–8110. 10.3892/mmr.2018.8927 29693132 PMC5983987

[B121] Wang J. Q. Cui Q. Lei Z. N. Teng Q. X. Ji N. Lin L. (2021). Insights on the structure-function relationship of human multidrug resistance protein 7 (MRP7/ABCC10) from molecular dynamics simulations and docking studies. Medcomm (Beijing) 2, 221–235. 10.1002/mco2.65 PMC8491190 34766143

[B122] Wang J. Q. Wu Z. X. Yang Y. Teng Q. X. Li Y. D. Lei Z. N. (2021). ATP-binding cassette (ABC) transporters in cancer: A review of recent updates. J. Evid-Based Med. 14, 232–256. 10.1111/jebm.12434 34388310

[B123] Wang J. Q. Yang Y. Cai C. Y. Teng Q. X. Cui Q. Lin J. (2021). Multidrug resistance proteins (MRPs): Structure, function and the overcoming of cancer multidrug resistance. Drug resist. Update. 54, 100743. 10.1016/j.drup.2021.100743 33513557

[B124] Wang J. Wang J. Q. Cai C. Y. Cui Q. Yang Y. Wu Z. X. (2020). Reversal effect of ALK inhibitor NVP-TAE684 on ABCG2-overexpressing cancer cells. Front. Oncol. 10, 228. 10.3389/fonc.2020.00228 32175279 PMC7056829

[B125] Wang Q. Li C. Zhu Z. Teng Y. Che X. Wang Y. (2016). miR-155-5p antagonizes the apoptotic effect of bufalin in triple-negative breast cancer cells. Anti-Cancer Drug 27, 9–16. 10.1097/CAD.0000000000000296 26398931

[B126] Wang Y. Lonard D. M. Yu Y. Chow D. C. Palzkill T. G. Wang J. (2014). Bufalin is a potent small-molecule inhibitor of the steroid receptor coactivators SRC-3 and SRC-1. Cancer Res. 74, 1506–1517. 10.1158/0008-5472.CAN-13-2939 24390736 PMC3947477

[B127] Wen L. Huang Y. Xie X. Huang W. Yin J. Lin W. (2014). Anti-inflammatory and antinociceptive activities of bufalin in rodents. Mediat. Inflamm. 2014, 171839. 10.1155/2014/171839 PMC3955582 24719521

[B128] Wettasinghe A. P. Singh N. Starcher C. L. DiTusa C. C. Ishak-Boushaki Z. Kahanda D. (2021). Detecting attomolar DNA-damaging anticancer drug activity in cell lysates with electrochemical DNA devices. Acs Sensors 6, 2622–2629. 10.1021/acssensors.1c00365 34156840 PMC8645337

[B129] Wu I. C. Chen Y. K. Wu C. C. Cheng Y. J. Chen W. C. Ko H. J. (2016). Overexpression of ATPase Na+/+ transporting alpha 1 polypeptide, ATP1A1, correlates with clinical diagnosis and progression of esophageal squamous cell carcinoma. Oncotarget 7, 85244–85258. 10.18632/oncotarget.13267 27845894 PMC5356733

[B130] Wu Z. X. Teng Q. X. Yang Y. Acharekar N. Wang J. Q. He M. (2022). MET inhibitor tepotinib antagonizes multidrug resistance mediated by ABCG2 transporter: *In vitro* and *in vivo* study. Acta Pharm. Sin. B 12, 2609–2618. 10.1016/j.apsb.2021.12.018 35646541 PMC9136566

[B131] Xie C. M. Chan W. Y. Yu S. Zhao J. Cheng C. H. (2011). Bufalin induces autophagy-mediated cell death in human colon cancer cells through reactive oxygen species generation and JNK activation. Free Radic. Bio. Med. 51, 1365–1375. 10.1016/j.freeradbiomed.2011.06.016 21763418

[B132] Xie J. T. Dey L. Wu J. A. Lowell T. K. Yuan C. S. (2001). Cardiac toxicity of resibufogenin: Electrophysiological evidence. Acta Pharmacol. Sin. 22, 289–297. 11742580

[B133] Xing Y. Lin N. U. Maurer M. A. Chen H. Mahvash A. Sahin A. (2019). Phase II trial of AKT inhibitor MK-2206 in patients with advanced breast cancer who have tumors with PIK3CA or AKT mutations, and/or PTEN loss/PTEN mutation. Breast Cancer Res. 21, 78. 10.1186/s13058-019-1154-8 31277699 PMC6612080

[B134] Xu Y. Liu X. Schwarz S. Hu L. Guo D. Gu Q. (2016), Inhibitory efficacy of bufadienolides on Na+,K+-pump activity versus cell proliferation. Biochem. Biophys. Rep. 6, 158–164. 10.1016/j.bbrep.2016.03.015 28955873 PMC5600443

[B135] Xu Y. Liu Y. Liu Q. Lu S. Chen X. Xu W. (2021). Co-delivery of bufalin and nintedanib via albumin sub-microspheres for synergistic cancer therapy. J. Control. Release 338, 705–718. 10.1016/j.jconrel.2021.08.049 34481023

[B136] Yan S. Qu X. Xu C. Zhu Z. Zhang L. Xu L. (2012a). Down-regulation of Cbl-b by bufalin results in up-regulation of DR4/DR5 and sensitization of TRAIL-induced apoptosis in breast cancer cells. J. Cancer Res. Clin. 138, 1279–1289. 10.1007/s00432-012-1204-4 PMC11824588 22447040

[B137] Yan S. Qu X. Xu L. Che X. Ma Y. Zhang L. (2014). Bufalin enhances TRAIL-induced apoptosis by redistributing death receptors in lipid rafts in breast cancer cells. Anti-Cancer Drug 25, 683–689. 10.1097/CAD.0000000000000095 24710190

[B138] Yang Q. Zhou X. Zhang M. Bi L. Miao S. Cao W. (2015). Angel of human health: Current research updates in toad medicine. Am. J. Transl. Res. 7, 1–14. 25755824 PMC4346519

[B139] Ye M. Tang Y. He J. Cao X. Liu J. Kou S. (2022). Alisol B 23-acetate increases the antitumor effect of bufalin on liver cancer through inactivating wnt/*β*-catenin Axis. Comput. Math. Method M. 2022, 6249534. 10.1155/2022/6249534 PMC9106498 35572840

[B140] Yu L. Chen Y. Tooze S. A. (2018). Autophagy pathway: Cellular and molecular mechanisms. Autophagy 14, 207–215. 10.1080/15548627.2017.1378838 28933638 PMC5902171

[B141] Yu Z. Guo W. Ma X. Zhang B. Dong P. Huang L. (2014). Gamabufotalin, a bufadienolide compound from toad venom, suppresses COX-2 expression through targeting IKKβ/NF-κB signaling pathway in lung cancer cells. Mol. Cancer 13, 203. 10.1186/1476-4598-13-203 25175164 PMC4161895

[B142] Yuan X. F. Tian H. Y. Li J. Jin L. Jiang S. T. Liu K. W. (2014). Synthesis of bufalin derivatives with inhibitory activity against prostate cancer cells. Nat. Prod. Res. 28, 843–847. 10.1080/14786419.2014.881363 24484199

[B143] Yuan Z. Liu C. Sun Y. Li Y. Wu H. Ma S. (2022). Bufalin exacerbates Photodynamic therapy of colorectal cancer by targeting SRC-3/HIF-1α pathway. Int. J. Pharm. 624, 122018. 10.1016/j.ijpharm.2022.122018 35839982

[B144] Zaretsky J. M. Garcia-Diaz A. Shin D. S. Escuin-Ordinas H. Hugo W. Hu-Lieskovan S. (2016). Mutations associated with acquired resistance to PD-1 blockade in melanoma. New Engl. J. Med. 375, 819–829. 10.1056/NEJMoa1604958 27433843 PMC5007206

[B145] Zhai B. Hu F. Yan H. Zhao D. Jin X. Fang T. (2015). Bufalin reverses resistance to sorafenib by inhibiting Akt activation in hepatocellular carcinoma: The role of endoplasmic reticulum stress. Plos One 10, e0138485. 10.1371/journal.pone.0138485 26381511 PMC4575108

[B146] Zhakeer Z. Hadeer M. Tuerxun Z. Tuerxun K. (2017). Bufalin inhibits the inflammatory effects in asthmatic mice through the suppression of nuclear factor-kappa B activity. Pharmacology 99, 179–187. 10.1159/000450754 28049205

[B147] Zhang C. Fu L. (2017). Effects of bufalin combined with doxorubicin on the proliferation and apoptosis of human lung cancer cell line A549 *in vitro* . Zhong Nan Da Xue Xue Bao Yi Xue Ban. 42, 762–768. 10.11817/j.issn.1672-7347.2017.07.004 28844998

[B148] Zhang D. M. Liu J. S. Deng L. J. Chen M. F. Yiu A. Cao H. H. (2013). Arenobufagin, a natural bufadienolide from toad venom, induces apoptosis and autophagy in human hepatocellular carcinoma cells through inhibition of PI3K/Akt/mTOR pathway. Carcinogenesis 34, 1331–1342. 10.1093/carcin/bgt060 23393227

[B149] Zhang X. Huang Q. Wang X. Xu Y. Xu R. Han M. (2017). Bufalin enhances radiosensitivity of glioblastoma by suppressing mitochondrial function and DNA damage repair. Biomed. Pharmacother. 94, 627–635. 10.1016/j.biopha.2017.07.136 28787697

[B150] Zhang X. Zhao X. Liu K. Che Y. Qiu X. Qu Y. (2020). Bufalin: A systematic review of research hotspots and antitumor mechanisms by text mining and bioinformatics. Am. J. Chin. Med. 48, 1633–1650. 10.1142/S0192415X20500810 33148004

[B151] Zhao H. Zhao D. Jin H. Li H. Yang X. Zhuang L. (2016). Bufalin reverses intrinsic and acquired drug resistance to cisplatin through the AKT signaling pathway in gastric cancer cells. Mol. Med. Rep. 14, 1817–1822. 10.3892/mmr.2016.5426 27357249

[B152] Zheng Y. Deng L. Cao H. Xu N. Zhang D. Tian H. (2022). Screening of bufadienolides from toad venom identifies gammabufotalin as a potential anti-inflammatory agent. Planta Med. 88, 43–52. 10.1055/a-1248-2626 33049786

[B153] Zhou H. Li Q. Xu C. Liang H. Wang Y. Duan Y. (2020). Prognosis of stage III cervical cancer: A two-way outcome study. Transl. Cancer Res. 9, 2565–2575. 10.21037/tcr.2020.02.70 35117616 PMC8798898

[B154] Zhu Z. Sun H. Ma G. Wang Z. Li E. Liu Y. (2012). Bufalin induces lung cancer cell apoptosis via the inhibition of PI3K/Akt pathway. Int. J. Mol. Sci. 13, 2025–2035. 10.3390/ijms13022025 22408435 PMC3292004

